# Functional mutagenesis screens reveal the ‘cap structure’ formation in disulfide-bridge free TASK channels

**DOI:** 10.1038/srep19492

**Published:** 2016-01-22

**Authors:** Matthias Goldstein, Susanne Rinné, Aytug K. Kiper, David Ramírez, Michael F. Netter, Daniel Bustos, Beatriz Ortiz-Bonnin, Wendy González, Niels Decher

**Affiliations:** 1 Institute for Physiology and Pathophysiology, University of Marburg, 35037 Marburg, Germany; 2 Center for Bioinformatics and Molecular Simulation, University of Talca, Talca, Chile

## Abstract

Two-pore-domain potassium (K_2P_) channels have a large extracellular cap structure formed by two M1-P1 linkers, containing a cysteine for dimerization. However, this cysteine is not present in the TASK-1/3/5 subfamily. The functional role of the cap is poorly understood and it remained unclear whether K_2P_ channels assemble in the domain-swapped orientation or not. Functional alanine-mutagenesis screens of TASK-1 and TRAAK were used to build an *in silico* model of the TASK-1 cap. According to our data the cap structure of disulfide-bridge free TASK channels is similar to that of other K_2P_ channels and is most likely assembled in the domain-swapped orientation. As the conserved cysteine is not essential for functional expression of all K_2P_ channels tested, we propose that hydrophobic residues at the inner leaflets of the cap domains can interact with each other and that this way of stabilizing the cap is most likely conserved among K_2P_ channels.

Two-pore domain potassium (K_2P_) channels have four transmembrane domains (M1 to M4) containing two pore loops per subunit^1^. The functional channels are formed by an antiparallel dimeric assembly, so that four pore loops form the selectivity filter of the channel^2^,^3^,^4^. Crystallization of K_2P_ channels revealed a unique cap structure formed by the two large extracellular linkers from the M1 to the pore loop (M1-P1 linker)^2^,^3^,^4^. The cap structure forms two tunnel-like side portals which serve as extracellular ion pathway (EIP) after the selectivity filter^5^. These side portals also play an important role in regulating the extracellular pH dependence of TASK channels^5^. Interestingly, following the initial crystal structure of TRAAK^2^, a second structure with higher resolution showed a domain-swapped chain connectivity of the M1-P1 linker at the helical cap which also results in structural rearrangement of two opposing transmembrane helices^3^. However, whether most of the TRAAK channels or only a fraction of the functional channels are domain-swapped still remained elusive, although the recently released TREK-1 (PDB ID: 4TWK) and TREK-2 6 structures were also crystallized in the domain-swapped orientation. Thus, a study providing information about the function of the cap and experiments providing insights towards the physiological orientation of the assembled channels would be very valuable.

In 1996 it was suggested by Lesage *et al*., that a K_2P_ channel dimer is covalently assembled by a disulfide-bridge and that the extracellular domain as well as the cysteine residues are essential elements for the dimerization process of TWIK-1 7. Crystallization of TWIK-1, TRAAK and TREK-2 confirmed the presence of a disulfide-bridge in homomeric K_2P_ channels^2^,^4^,^6^. In addition, TASK-2, TASK-4, TALK-1, TREK-1, TRESK, KCNK7 and TWIK-2 share a homologous cysteine residue. However, for some K_2P_ channels there is evidence that the disulfide bond is not required for dimerization or the functional expression of homodimers^8^,^9^,^10^. Accordingly, other unidentified mechanisms may exist for the functional assembly of K_2P_ channels, especially as the TASK-1/3/5 and THIK-1/2 subfamilies do not contain a homologous cysteine residue in the M1-P1 linker. Unfortunately, no ‘disulfide-bridge free’ K_2P_ channel could be crystallized yet. The lack of a cysteine in the M1-P1 linker, together with a very strong computational coiled-coil prediction in this domain of TASK channels, prompted us to study the role of this linker in TASK-1 channels. In the current study, using functional alanine-mutagenesis screens, we have built an experimentally validated model of the disulfide-bridge free cap structure of the TASK-1 channels in the domain-swapped orientation. Combined with a mutagenesis screen in TRAAK our data suggest that the cysteine residues in the cap structure are not relevant for the functional expression of K_2P_ channels. Furthermore, our model indicates that the cap structure formation of TASK channels is conserved to that of K_2P_ channels containing an extracellular disulfide-bridge.

## Results

### Potential coiled-coil domain in the cysteine-free M1-P1 linker of TASK-1 channels

**Figure 1:**
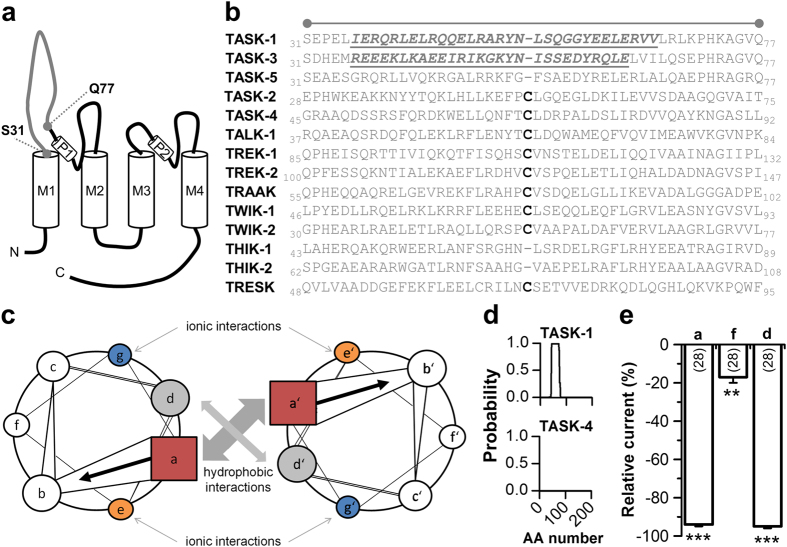
A coiled-coil prediction in the M1-P1 linker of TASK-1 channels. (**a**) Transmembrane topology of TASK-1. The extracellular region corresponding to the cap structure of other K_2P_ channels is indicated. (**b**) Amino acid sequence alignment of different K_2P_ channels for the extracellular region indicated in (**a**). The cysteine residues, conserved in most K_2P_ channels, are highlighted in bold. Residues of the four heptad repeat coiled-coil predictions in TASK-1 and TASK-3 are indicated in italics and by underlining. (**c**) Coiled-coil structures usually contain a repeated pattern of hydrophobic (h) and charged (c) amino-acid residues. ‘hxxhcxc’ refers to a heptad repeat re-occuring after every two turns of the helix. The positions within the heptad repeat are commonly labeled as ‘a–g’, where ‘a’ (*red*) and ‘d’ (*gray*) are the hydrophobic core positions, often occupied by the amino acids isoleucine, leucine, or valine, thus stabilizing helix dimerization through hydrophobic and van-der-Waals interactions, whereas ‘e’ (*orange*) and ‘g’ (*blue*) are typically solvent-exposed, polar residues (e.g. glutamate or lysine) that give specificity between the two helices through interhelical electrostatic interactions. The remaining three positions (‘b’, ‘c’, and ‘f’, *white*) must be all hydrophilic, as these will form helical solvent exposed surfaces. (**d**) Coiled-coil prediction tools revealed with a high probability a four-fold coiled-coil repeat in TASK-1 (*top*), whereas for instance in TASK-4 no coiled-coil domain was predicted (*bottom*). AA, amino acid. (**e**) Typical interaction sites of coiled-coil domains were mutated and current amplitudes, analyzed at +40 mV, were compared to wild-type TASK-1. ‘a’, indicates the four-fold mutation of all a-sites (R38A/Q45A/Y52A/Y59A); ‘f’, indicates the four-fold mutation of all f-sites (L43A/A50V/G57A/R64A); ‘d’, indicates the four-fold mutation of all d-sites (L41A/L48A/S55A/L62A). **p < 0.01; ***p < 0.001. Unpaired Student’s T-Test. Data are presented as Mean ± SEM. The number of experiments are included in the bar graph.

In 1996 Lesage *et al*. proposed that the extracellular M1-P1 linker of K_2P_ channels is essential for the dimerization process and that channel dimers are covalently assembled via a disulfide-bridge within this linker^7^. Most K_2P_ channels share a homologues cysteine residue, while TASK-1, TASK-3, TASK-5, THIK-1 and THIK-2 do not have a cysteine in the M1-P1 linker (Fig. 1a,b). Before the first K_2P_ channels were crystallized, we aimed at understanding the structural differences between the particularly long extracellular M1-P1 linkers of K_2P_ channels. Using secondary structure predictions of the M1-P1 linkers, we identified a strong coiled-coil prediction (Expasy, COILS version 2.1 11) which was very prominent in the non disulfide-bridge containing TASK-1 and TASK-3 channels. The coiled-coil domain is a common structural motif, consisting of two to five α-helices mutually wrapped into a left-handed helix forming a supercoil^12^ (Fig. 1c; Supplementary Fig. S1a). Interestingly, only for TASK-1 and TASK-3 strong 4-fold heptad repeat predictions were observed (Supplementary Fig. S1b). Figure 1d illustrates examples of 4-fold heptad repeat coiled-coil predictions for TASK-1 (no cysteine in the M1-P1 linker) and TASK-4 (cysteine containing M1-P1 linker). To functionally probe for a coiled-coil domain, we introduced mutations in TASK-1 affecting all ‘a’-sites (R38A/Q45A/Y52A/Y59A) and all ‘d’- sites (L41A/L48A/S55A/L62A) which should be relevant for the coiled-coil formation (Fig. 1e; Supplementary Fig. S1b), or all ‘f’-sites which should be not relevant for coiled-coil assembly (L43A/A50V/G57A/R64A) (Fig. 1e; Supplementary Fig. S1b). Subsequently, we studied the current amplitudes of the four-fold mutations using two-electrode voltage clamp (TEVC) recordings in *Xenopus* oocytes. Consistent with a coiled-coil domain in the extracellular M1-P1 linker of TASK-1, we revealed a strong current reduction for the ‘a’-site mutant (93%) as well as for the ‘d’-site mutant (93%), whereas the ‘f’-site mutant showed only a minor current reduction (20%) (Fig. 1e). These experiments prompted us to alanine-scan the extracellular M1-P1 linker to identify amino acids relevant for the dimerization or functional expression of TASK-1 channels.

### Functional alanine-scanning of the extracellular M1-P1 linker of TASK-1

**Figure 2:**
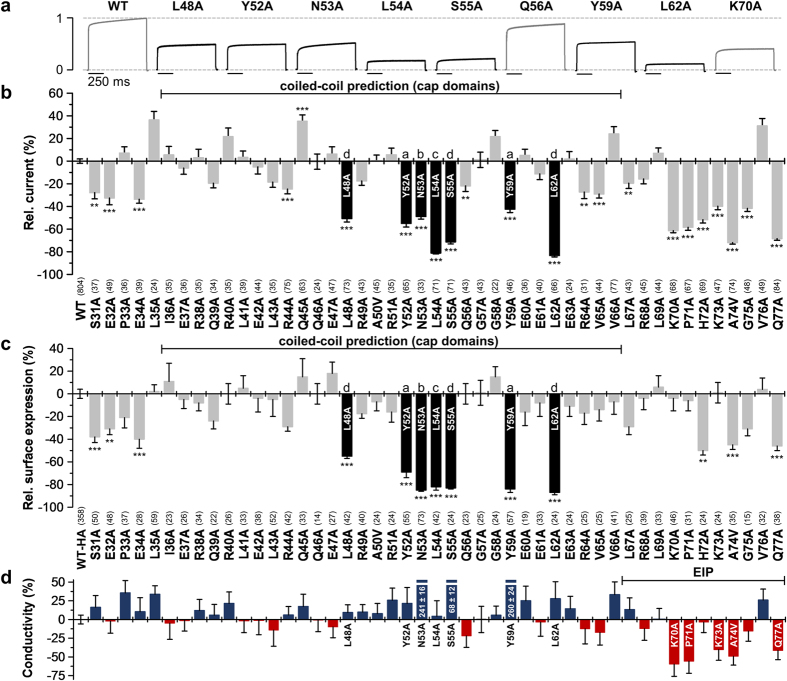
Alanine scan through the extracellular M1-P1 linker of human TASK-1 reveals hydrophobic residues relevant for functional surface expression. (**a**) Normalized representative current traces of wild-type and mutant TASK-1 channels recorded in *Xenopus* oocytes by a voltage step to +40 mV. (**b**) Relative current amplitudes at +40 mV compared to wild-type TASK-1 (WT). Mutants within the predicted coiled-coil domain exhibiting a pronounced current amplitude reduction are highlighted in *black*. (**c**) Relative surface expression analyzed with a chemiluminescence assay of wild-type TASK-1 with an extracellular HA epitope (WT-HA) and TASK-1 mutants introduced into the WT-HA background. Mutants within the predicted coiled-coil domain showing a strong reduction in relative surface expression are highlighted in *black*. (**b**,**c**) **p < 0.01; ***p < 0.001. Unpaired Student’s T-Test. Significance was probed against WT or WT-HA. Data are presented as Mean ± SEM. The number of experiments are indicated in parentheses above the construct name. (**d**) The ‘conductivity’ of TASK-1 mutants was calculated by dividing the relative change in current amplitudes of a mutant by the relative change in surface expression of the respective mutant. Red bars indicate a loss of channel gating, due to a reduced single-channel conductance or open probability (Po). Data are presented as Mean ± SEM.

The alanine scan ranged from S31 up to Q77 (Figs 1a,b and 2), including the 31 amino acid span of the coiled-coil prediction (Figs 1b and 2). Alanine residues were mutated to valine. For all mutants current amplitudes were analyzed after expression in *Xenopus* oocytes. Representative current traces resulting from a voltage step to +40 mV are depicted in Fig. 2a. While the alanine-scan revealed only a few mutants with a significant current increase, several mutations with a strong current reduction were observed (Fig. 2a,b). Mutants located in the predicted coiled-coil region exhibiting a very strong current decrease (L48A, Y52A, N53A, L54A, S55A, Y59A and L62A) are highlighted as black bars (Fig. 2b). Interestingly, within this span of the coiled-coil prediction five of these seven residues highly relevant for functional channel expression were located at hydrophobic ‘a’- and ‘d’-sites (L48, Y52, S55, Y59 and L62) which might be relevant for hydrophobic coiled-coil formation (Fig. 2b; Supplementary Fig. S1b). However, the residues identified essential for functional channel expression were not strictly following a coiled-coil prediction, as not all ‘a’- and ‘d’-sites were affected and ionic ‘e’- and ‘g’-sites remained normal. In addition, N53A and L54A were located at ‘b’- and ‘c’-sites. Accordingly, mutating all the residues we have identified to alanine (L48A/Y52A/N53A/L54A/S55A/Y59A/L62A) did not destroy the *in silico* coiled-coiled prediction (p = 0.999). Thus, despite the initial computational predictions it is not a classical coiled-coil domain stabilizing the extracellular structure of this disulfide-bridge free K_2P_ channel. In contrast, alignments with the TWIK, TRAAK and TREK channels^2^,^3^,^4^,^6^-crystallized in the progress of this study - suggest that the coiled-coil prediction and the containing loss-of-function mutations in L48, Y52, N53, L54, S55, Y59 and L62, directly fall into the region of the two extracellular cap domains, while the residues following the coiled-coil prediction (starting from amino acid L67) are more likely to be homologous to the previously described EIP^5^.

### Loss-of-function mutations within the cap domains are caused by a decreased surface expression

To test for surface expression of the different alanine mutants, we performed an ELISA based chemiluminescence assay in *Xenopus* oocytes. TASK-1 and all alanine-mutants of the M1-P1 linker were tagged with an hemagglutinin (HA) epitope near the end of the extracellular P2-M4 linker. The relative surface expression compared to TASK-1 wild-type (WT-HA) is shown in Fig. 2c. Interestingly, the current reduction observed for mutations within the cap (L48A, Y52A, N53A, L54A, S55A, Y59A, L62A) was paralleled by a reduction in surface expression (Fig. 2b versus Fig. 2c), indicating a reduced functional expression at the surface as a mechanism for the strong loss-of-function. To probe for changes in the channel gating, we analyzed the ratio of the current amplitudes (Fig. 2b) to surface expression (Fig. 2c) as a measure for the ‘conductivity’ of the mutants (Fig. 2d). Note that the changes in ‘conductivity’ observed here can either result from an altered single-channel conductance or open probability, which was not studied in further detail. These analyses confirmed that L48A, Y52A, N53A, L54A, S55A, Y59A and L62A have a normal ‘conductivity’ (Fig. 2d), while functional expression at the surface membrane is reduced (Fig. 2c).

### Loss-of-function mutations in the region following the cap domains (putative EIP) are caused by a decreased ‘conductivity’

Alanine mutations in the region following the putative cap domains, in the range of K70 to Q77, are very prone to induce a loss-of-function (Fig. 2b). This region is likely to form the previously described EIP in TASK channels^5^. However, in contrast to the seven mutations within the putative cap domains (Fig. 2c,d), only H72A caused a reduced surface expression while retaining a normal channel gating (Fig. 2c,d). The other loss-of-function mutations in the putative EIP were caused by a combination of a reduced surface expression and a reduced conductivity (A74V, Q77A) (Fig. 2c,d) or by a pure gating defect (K70A, P71A, K73A), as these channels reached the plasma membrane with a normal efficiency but the current amplitudes are strongly reduced (Fig. 2c,d). This loss of ‘conductivity’ which we have observed in this region by alanine-mutations (Fig. 2d) is in good agreement with the idea that the hydrophilicity in the EIP is important for the conductivity of TASK channels^5^. Thus, we propose that the mutations in the EIP change the conductivity of the channel either due to changes in the single-channel conductance or the open probability, while the mutations within the cap might be important for the channel assembly or dimerization.

### Impaired surface expression of ‘hit’ mutations in the cap domains is caused by a reduced abundance of dimeric TASK-1 protein

**Figure 3:**
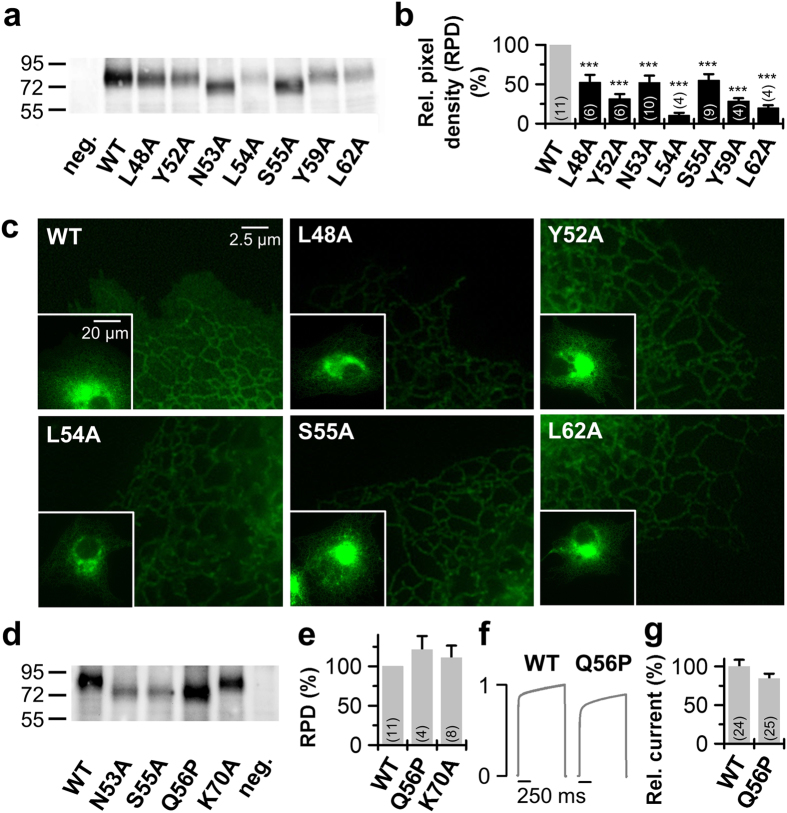
Protein expression of the identified TASK-1 ‘hits’. (**a**) Western blot analysis of *Xenopus* protein lysates after injection of HA-tagged TASK-1 wild-type (WT) or indicated TASK-1 mutants using anti-HA antibodies. Samples were not treated with a reducing agent as DTT, thus the proteins appear as a dimer. (**b**) Analysis of the pixel intensities of the protein signals corresponding to dimeric TASK-1 from 4 to 11 independent western blots normalized to TASK-1 wild-type without DTT. Image J was used for pixel intensity analysis. RPD: relative pixel density. (**c**) Live cell imaging of TASK-1 mutants in HeLa cells. HeLa cells were transfected with the indicated pEGFP-TASK-1 mutants and fluorescence imaging was performed 48 h after transfection. (**d**) Western blot analysis of protein lysates of oocytes 48 hours after injection of HA-tagged TASK-1 wild-type (WT) or indicated TASK-1 mutants using anti-HA antibodies. Samples were not treated with a reducing agent as DTT, thus proteins appear as a dimer. (**e**) Analysis of the pixel intensities of the protein signals corresponding to dimeric TASK-1 from 4 to 11 independent western blots normalized to TASK-1 wild-type without DTT. Image J was used for pixel intensity analysis. (**f**) Current traces of TASK-1 WT compared to the Q56P mutant, preventing glycosylation. (**g**) Current amplitudes at +40 mV for TASK-1 (WT) and the Q56P mutant normalized to TASK-1 were plotted. (**b**,**e**,**g**) ***p < 0.001. Unpaired Student’s T-Test. Significance was probed against wild-type TASK-1 (WT). Data are presented as Mean ± SEM. The number of experiments are included in the bar graphs.

To probe whether the reduced surface expression of mutations in the cap was caused by an impaired channel dimerization, we performed western blot experiments injecting equal amounts of HA-tagged constructs into oocytes (Fig. 3a). Analyzing the amount of dimeric TASK-1 protein for L48A, Y52A, N53A, L54A, S55A, Y59A and L62A, we found that all these ‘hits’, identified in the cap structure, showed a strongly reduced expression of dimeric protein compared to wild-type TASK-1 (Fig. 3a,b). This reduction was not exclusively present for the dimeric protein (Fig. 3a,b; Supplementary Fig. S2), suggesting that the diminished formation of stable TASK-1 protein is the most likely cause for the impaired functional expression at the cell surface. As a control we used the K70A mutation, as this amino acid exchange is located in the putative EIP and should not interfere with channel dimerization. In contrast to the mutations within the cap domains, the K70A mutation has a normal surface expression (Fig. 2c) and a pure loss-of-function by a reduced ‘conductivity’ (Fig. 2b,d). Consistently, this mutation had a normal abundance of dimeric and monomeric protein (Fig. 3d,e; Supplementary Fig. S2).

TASK-1 channels studied with fluorescence imaging were primarily localized at the ER and only a weak surface expression was detected (Fig. 3c), similar as previously described^13^. Notably, for the wild-type TASK-1 surface expression appears as uniform staining of the entire cell, also between the ER^13^, which is not present for the mutants (Fig. 3c). Consistent with the reduced functional expression of the mutants at the surface membrane in oocytes (Fig. 2b,c), and an impaired protein stability, we observed a preferential fluorescence of the mutations to an intracellular compartments. As misfolded proteins are known to accumulate in the ER and concomitantly cause ER stress, the TASK-1 mutations with diminished protein stability (Fig. 3b) were primarily located in a compartment most probably reflecting the endoplasmic reticulum (Fig. 3c).

### The impaired surface expression of N53A and S55A, by a reduced abundance of dimeric TASK-1 channels, is not caused by an altered glycosylation

The residue N53 resides at the homologues position to the cysteine at the tip of the TWIK-1, TREK-2 and TRAAK cap structure (Fig. 1b). This asparagine is predicted as a weak glycosylation site and S55 is part of the consensus motif for a glycosylation at position N53. In fact, the western blot analyses revealed that the molecular weight of N53A and S55A is reduced by approximately 10 kDa (Fig. 3a) which supports that TASK-1 channels might be glycosylated at N53. To show that the loss-of-function by N53A and S55A is caused by interfering with the channel structure or assembly and is not indirectly caused by an altered glycosylation, we next studied the mutation Q56P. The Q56A mutation showed normal current amplitudes and surface expression, thus Q56 is not an essential structural requirement for the assembly of the dimeric cap structure. Introducing a proline after the N-(x)-S motif destroys the glycosylation site^14^. Thus, with a Q56P mutation it is possible to destroy the glycosylation site at N53 without interfering with the structural requirements of having an asparagine at position 53 and a serine at position 55. The Q56P mutation had a similar reduction in molecular weight as N53A and S55A (Fig. 3d), indicating the expected loss of glycosylation at position N53. However, unlike for N53A and S55A the abundance of dimeric protein was not reduced (Fig. 3d,e) and the functional expression was not altered (Fig. 3f,g). These data indicate that the reduced abundance of dimeric proteins by N53A and S55A and the resulting loss of functional expression is not caused by an altered glycosylation at position N53. Summarizing, the residues L48A, Y52A, N53A, L54A, S55A, Y59A and L62A might be the major prerequisite for the stability and dimerization of the cap structures in TASK-1 channels.

### Residues L48A, Y52A, L54A, S55A, Y59A and L62A are involved in intersubunit interactions with the closely related TASK-3 channels

**Figure 4:**
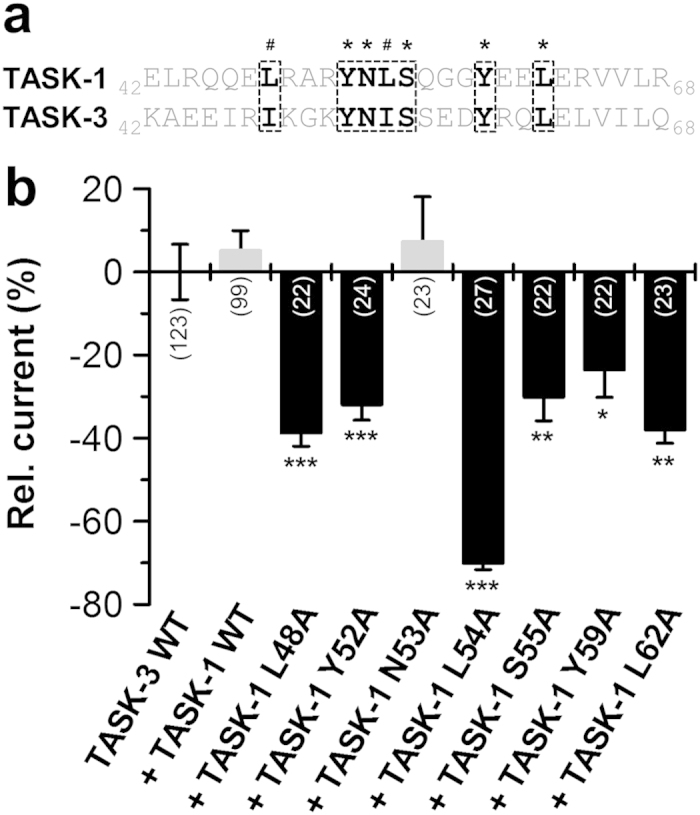
Co-expression of TASK-1 ‘hits’ from the alanine scan with TASK-3. (**a**) Sequence alignment of the tip of the TASK-1 and TASK-3 cap. TASK-1 ‘hits’ are highlighted in bold. *conserved residues; ^#^conservative amino acids. (**b**) TASK-3 was injected in *Xenopus* oocytes alone (TASK-3 WT), in the presence of TASK-1 WT or in presence of TASK-1 mutants. Changes in current amplitudes compared to TASK-3 WT were analyzed at +40 mV. *p < 0.05; **p < 0.01; ***p < 0.001. Unpaired Student’s T-Test. Significance was probed against TASK-3 WT. Data are presented as Mean ± SEM. The number of experiments are included in the bar graphs.

A sequence alignment of the M1-P1 linkers of TASK-1 and TASK-3 revealed a high conservation of the critical amino acids identified in TASK-1 (Fig. 4a). To elucidate a functional conservation of the cap structures and a role of these residues for hetero dimerization, we expressed wild-type TASK-3 in the absence and presence of the identified TASK-1 mutants (Fig. 4b). In all cases (except for N53A) the co-expression of the TASK-1 mutants reduced the current amplitude of wild-type TASK-3. In contrast, co-expression of TASK-1 wild-type lead to a minor current increase. As the current suppression by the TASK-1 mutants was conferred to the co-expressed wild-type TASK-3 in a dominant-negative manner, the TASK-1 mutations must cause a disturbance in the heteromeric channel complex with TASK-3. Thus, the mutations might perturb intersubunit interactions of these conserved residues which are necessary for a stable heteromeric cap structure. Thus, it is likely that the residues L48A, Y52A, L54A, S55A, Y59A and L62A are also involved in intersubunit (not intrasubunit) interactions for homomeric TASK channel assembly.

### A domain-swapped TASK-1 cap structure model based on the functional mutagenesis screens

**Figure 5:**
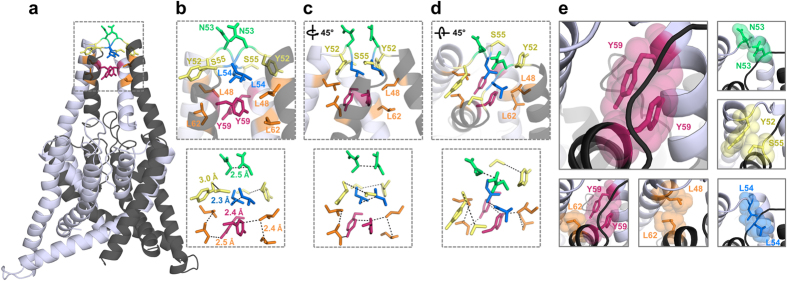
TASK-1 cap structure model based on domain-swapped TREK-2. TASK-1 cap structure illustrated after 100 ns of MD simulations. The two different subunits are shown in *gray* and *light gray*. TASK-1 cap model with the residues identified essential for channel expression, which are involved in intersubunit interactions. Pairs of residues that interact with each other are illustrated using the same color code. (**a**) Complete structure of the TASK-1 model. (**b**) Zoom-in to the boxed area of (**a**) illustrating interacting residues and their distances, and (**c**) after 45° rotation or (**d**) after additional tilting of 45° out of the plane. (**e**) Close-up in the cap on specific pairs of amino acids involved in intersubunit interactions.

**Figure 6:**
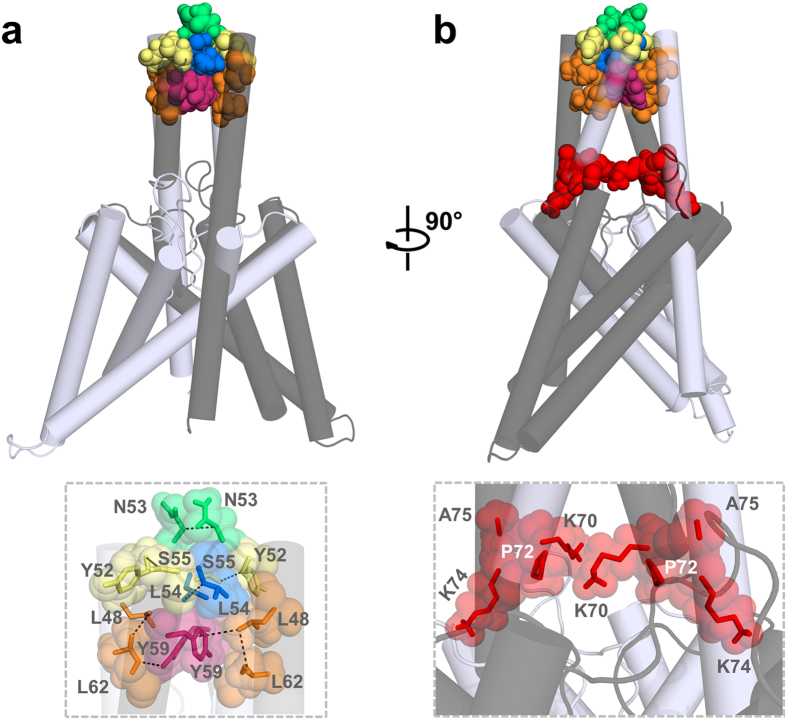
Hydrophobic interactions stabilize the tip of the TASK-1 cap structure. TASK-1 ‘hits’ identified in the alanine scan are highlighted in the TASK-1 homology model based on the domain-swapped TREK-2, following a 100 ns MD simulation. The two different subunits are shown in *gray* and *light gray*. (**a**) Residues stabilizing the tip of the cap structure are shown in space fill mode and a zoom-in is provided at the bottom. (**b**) 90° rotation of the model. Mutations resulting in a loss of ‘conductivity’ are illustrated in red space fill and a zoom-in showing these residues located in the EIP is provided at the bottom.

Next we generated homology models of the cysteine-free cap structure of TASK-1 based on the x-ray structures of TRAAK, domain-swapped TRAAK, TWIK-1 and TREK-2 2,3,4,6. After generating the homology models, we performed MD simulation of 10 ns duration analyzing putative intersubunit interactions that might stabilize the cap structure. Here the average distances of the residues, which we identified in the coiled-coil domain as relevant for channel dimerization and functional expression, were quantified over a time period of 10 ns (Table 1; Supplementary Tables S1–3). Interactions of key residue in the helical cap were evaluated measuring carbon-carbon atom distances (see also Methods). A relative remote cut-off of 6.0 Å (instead of 4 Å) was used to probe for interactions, as the predefined C atoms selected for the automatic distance measurements (Supplementary Fig. S3) do not necessarily reflect the closest C atom for carbon-carbon interactions and in addition the space of the H atoms are not considered. Using the TWIK-1 structure as a template for a homology model of the TASK-1 cap, the distances of the residues which we identified in our alanine mutagenesis screens, were overall too remote to contribute to an intersubunit interaction (Supplementary Table S1). Analyzing intersubunit distances within the cap structure, the homology model predicted only one putative intersubunit interaction involving our ‘hit’ residues (Y59 with Y59, Supplementary Table S1). Using TRAAK as template for the cap structure model, three intersubunit interactions (involving L48, N53, L54 and Y59) were predicted (Supplementary Fig. S4) and the intersubunit distances of our ‘hit’ residues were overall smaller, indicating that TRAAK was a more suitable template (Supplementary Table S2). Using the domain-swapped TRAAK crystal as a template, we identified three putative intersubunit interactions, involving L48, Y52, L54, S55 and L62 (Supplementary Table S3). However, the homology model which was in perfect agreement with our alanine-scanning mutagenesis was based on the domain-swapped TREK-2 crystal structure. This model had much smaller average distances for the residues relevant for dimerization and it predicted six intersubunit interactions (Table 1 and Fig. 5), confirming all the residues (L48, Y52, N53, L54, S55, Y59 and L62) we have identified in the alanine mutagenesis screens (Table 1; Fig. 2). Subsequently, this homology model, which was in full agreement with our functional data, was probed by additional 90 ns of unrestrained MD simulations to generate the final model of a K_2P_ channel with a cysteine-free cap structure (Fig. 5). During the entire 100 ns of MD simulations the cap structure and the identified interactions (Table 1) remained intact (see Methods). In this final model of the TASK-1 cap structure (Fig. 5a), N53, L54 and Y59 interact with the identical residues of the opposing subunit (Fig. 5b–e; see the diagonal in Table 1). In addition, L48 interacts with L62, Y52 with S55 and L62 with Y59 of the opposing subunit (Fig. 5b–e; under the diagonal in Table 1). Analyzing the smallest distance between these residues (not the automated measure of preset carbon-carbon atom distances (Supplementary Fig. S3) as in Table 1), all these residues were involved in hydrophobic interactions with a distance of less than 3 Å (Fig. 5b). Mutating Y59 to alanine, leucine, aspartate, glutamate, lysine, arginine or cysteine resulted in a loss-of-function, while a phenylalanine did not disturb functional expression (Supplementary Fig. S5), supporting our model that Y59 is involved in a π–π interaction with Y59 of the neighboring subunit (Fig. 5b,e). As mentioned above, it still remained an open question whether the cap structure of K_2P_ channels predominantly exist in the domain-swapped orientation or not. TRAAK crystallization revealed a domain-swapped and non domain-swapped structure whereas for TREK-2 only a domain-swapped structure has been described so far. Our functional data together with molecular modeling experiments are in perfect agreement with a domain-swapped cap structure of TASK-1 (Fig. 5). Furthermore our data indicate that hydrophobic interactions at the tip of the cap structure stabilize the dimeric channels (Fig. 6a,b), while residues that alter channel conductivity or gating are facing the EIP located above the selectivity filter (Fig. 6b).

### Functional conservation of hydrophobic interactions in the cap between TRAAK and disulfide-bridge free TASK-1 channels

**Figure 7:**
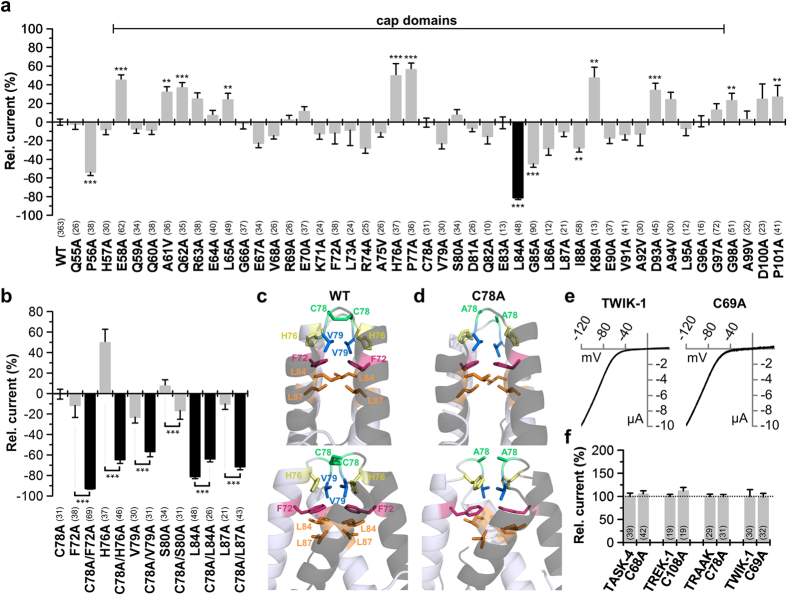
Alanine scan through the extracellular M1-P1 linker of human TRAAK. (**a**) Relative current amplitudes at +40 mV compared to TRAAK wild-type (WT). Mutations in the cap exhibiting a pronounced current amplitude reduction are highlighted in *black*. Note that the C78A mutation at the tip of the cap structure did not affect current amplitudes. (**b**) TRAAK mutations at the sites homologous to the TASK-1 ‘hits’ were functionally studied in TRAAK alone (as in (**a**)) or in the absence of the cysteine at position 78 (C78A). F72 in TRAAK corresponds to L48 in TASK-1, H76 to Y52, C78 to N53, V79 to L54, S80 to S55, L84 to Y59 and L87 to L62. Pronounced reduction in current amplitudes compared to the C78A mutant alone are illustrated in *black*. (**c**) Domain-swapped TRAAK cap structure after 10 ns of MD simulation illustrating the relevant residues identified in (**b**). Upper panel shows the orientation as in Brohawn *et al*., 2012 and the lower panel is rotated by 90°. (**d**) Domain-swapped TRAAK cap structure of the C78A mutation after 10 ns of MD simulation, illustrating the relevant residues identified in (**b**). *Upper panel* shows the orientation as in Brohawn *et al*., 2012 and the lower panel is rotated by 90°. (**e**) Representative current traces of TWIK-1 and TWIK-1 C69A recorded in oocytes, using a voltage ramp from −120 to +40 mV. (**f**) Changes in relative current amplitudes at +40 mV, after mutating the conserved cysteine residues in the cap structure of different K_2P_ channels. (**a**,**b**,**f**) **p < 0.01; ***p < 0.001. Significance was probed using unpaired Student’s T-Test against the respective wild-type channel, unless for panel (**b**), here significance of the double mutation was probed against the respective single mutation without C78A. Data are presented as Mean ± SEM. The number of experiments are given above the construct name or included in the bar graphs.

To probe for the functional difference in dimerization compared to a K_2P_ channel containing a disulfide-bridge, we performed a functional alanine-screen through the extracellular M1-P1 linker of TRAAK. Moreover this channel was crystallized in two cap variants, thus functional data might provide conclusions towards the most likely chain connectivity of the cap (domain-swapped or not). Unexpectedly, the alanine-scan in TRAAK identified only one lipophilic residue (L84) as functional relevant (Fig. 7a), while lipophilic residues were previously thought to form a hydrophobic zipper-like structure in the core of the helical cap domains, including residues L65, F72, L73, L84, L87, I88, V91, A92 and A94 2. The lack of functional relevance of this lipophilic zipper described by Brohwan *et al*., might be attributed to the fact that TRAAK channels are primarily stabilized by a disulfide-bridge between two cysteine residues (C78) at the tip of the cap structure, which are at the site homologous to N53 in TASK-1. Surprisingly, we found that the disulfide-bridge has no major relevance for the functional expression of the channels, as the C78A mutant had a regular functional expression (Fig. 7a). Thus, the TRAAK channels might be stabilized by a concerted action of the disulfide-bridge and a set of hydrophobic interactions. Accordingly, hydrophobic interactions in the C78A mutant might keep the channel functional in the absence of the disulfide-bridge. Therefore, we next mutated the residues F72, H76, V79, S80, L84 and L87, which correspond to the residues L48, Y52, L54, S55, Y59 and L62 we identified in TASK-1, in the presence of the C78A mutation (Fig. 7b). Studying TRAAK channels without cysteine (C78A mutant), we found that with the exception of S80 (corresponding to S55), all the homologous residues, identified in the TASK-1 mutagenesis screen, become highly relevant for functional expression of TRAAK (Fig. 7b). Figure 7c illustrates that the lipophilic residues, homologous to the TASK-1 ‘hits’, which stabilize the TRAAK channel are forming a zipper-like structure which resembles the model, we describe for the TASK-1 cap (Fig. 5). This lipophilic zipper was present and stable after 10 ns of MD simulations for the domain-swapped crystal structure (Fig. 7c), but also for an *in silico* mutated C78A TRAAK channel (Fig. 7d). These data suggest that the lipophilic zipper is present in the TRAAK cap and is able to stabilize the channel in the absence of the disulfide-bridge. However, Brohawn *et al*., 2012 proposed that the residues L65, L73, P77, I88, V91, A92 and A94 might be relevant for the hydrophobic zipper and the structure of the cap. Therefore, we also studied these mutations in the C78A background (Supplementary Fig. S6). From these residues only I88 had a major relevance in the absence of the disulfide-bridge (Supplementary Fig. S6), confirming the predominant relevance of the residues at the sites we also identified in TASK-1. These functional data are unexpected when looking at the first TRAAK structure, as the residues L65, L73, P77, V91, A92 and A94 played no major role (Fig. 7a), even in the absence of the disulfide-bridge (Supplementary Fig. S6). In contrast and also unexpected, our functional mutagenesis screen revealed that the structures and the positions of the amino acids (with the exception of I88) stabilizing the caps of cysteine and non-cysteine containing K_2P_ channels are highly conserved.

As we have observed that the residues F72, H76, V79, L84, L87 and I88 are relevant for a stable TRAAK expression, we next analyzed whether these residues are more likely to stabilize a domain-swapped or a non domain-swapped cap structure (Supplementary Fig. S7; Supplementary Tables S4–5). Despite some differences at the tip of the cap structure these residues appear in both TRAAK structures to be equally important in terms of forming a hydrophobic interaction domain which stabilizes the cap (Supplementary Fig. S7). Therefore, our functional data does not favor the physiological predominance of a particular cap structure variant, which is also in agreement with the crystallization of both, the domain-swapped and non domain-swapped, TRAAK channel.

### Disulfide-bridges in the cap structure are mostly not essential for the functional expression of K_2P_ channels

As expected performing western blot analysis of TASK-1, TASK-3 and TWIK-1 under non-reducing conditions the molecular weight of the major band are corresponding to the dimeric channel protein (Supplementary Fig. S8). Unexpectedly despite the lack of a cysteine in the M1-P1 linker, DTT treatment resulted for TASK-1 and TASK-3 in an intensive band corresponding to the monomeric channel protein (Supplementary Fig. S8). For TASK-1 this behavior was observed independent of the tag used (EGFP or HA-epitope) and was similar to the effects observed for the cysteine containing TWIK-1 (Supplementary Fig. S8). Similar results were obtained using ß-mercapto-ethanol instead of DTT (data not shown). Thus, biochemically, we did not observe differences between channels containing a cysteine in the cap structure (TWIK-1) or not (TASK-1 and TASK-3). These data suggest that reducing agents like DTT or ß-mercapto-ethanol in combination with SDS might have denaturing effects that might have led to wrong conclusions about the functional relevance of disulfide-bridges in the cap structure.

As TWIK-1 channels were reported to dimerize via C69 in the cap structure and mutations at this site were reported to result in a loss of functional expression^7^, the western blot experiments were reproduced using the identical expression constructs and antibodies as in the original study by Lesage *et al*. However, the results still resembled the data from TASK-1 and TASK-3 (Supplementary Fig. S8), questioning the relevance of the disulfide-bridge in TWIK-1. TWIK-1 channels were previously difficult to record due to a small current amplitude^7^, however using Rb^+^ as a charge carrier, TWIK-1 generates large and stable macroscopic currents^15^,^16^. Recording TWIK-1 currents of the C69A mutation under these conditions revealed that the disulfide-bridge has no major relevance for the functional expression of TWIK-1 (Fig. 7e,f), similar as for TRAAK C78A (Fig. 7a,f). Next, we probed the functional relevance of mutating the putative disulfide-bridge of TASK-4 (C68A) and TREK-1 (C108A) and also here preventing the disulfide-bridge by mutagenesis did not alter the functional expression (Fig. 7f). Thus, despite the clear presence of a disulfide-bridge in the crystal structures of TRAAK, TREK-1 and TWIK-1 2,3,4, this covalent binding seems to be of minor relevance for the functional expression, indicating that the formation of a disulfide-bridge does not play a predominant role in the dimerization or functional expression of many K_2P_ channels.

## Discussion

The recent crystallization of K_2P_ channels revealed that two extracellular M1-P1 linkers of the dimeric channels form a cap which is at the tip stabilized by a disulfide-bridge^2^,^3^,^4^,^5^,^6^,^7^. This cysteine in the first extracellular loop is conserved in most K_2P_ channels. We aimed at understanding the structural differences between these channels and the K_2P_ channels that do not contain a disulfide-bridge in the extracellular cap, like TASK-1 and TASK-3. Functional mutagenesis screens together with the structures of crystallized K_2P_ channels as templates, were used to build an experimentally validated model of the disulfide-bridge free cap structure of TASK-1. Moreover, we alanine-screened the TRAAK M1-P1 linker to identify residues relevant for homomerization and additionally investigated the role of cysteine residues in the M1-P1 loop of other K_2P_ channels. Our data suggest that the cysteine residues in the cap structure of K_2P_ channels are less important for channel assembly than expected. In particular for TASK-1, using a domain-swapped K_2P_ channel crystal structure as a template for the modeling, we can show that the cap is stabilized by a defined set of hydrophobic intersubunit interactions which are primarily present near the tip of the cap. Our data indicate that TASK-1 channels might primarily assemble in the domain-swapped orientation, and our functional data obtained with TRAAK could be reconciled with the existence of both channel variants crystallized.

Comparing our data of the TASK model with the functional data in TRAAK channels which lack the extracellular disulfide-bridge (C78A mutant), revealed that the overall structure and the sites of hydrophobic interactions are conserved between cysteine-containing and cysteine-free cap structures of K_2P_ channels. This data is supported by our observation that the cysteine residues also do not play a role for the functional expression of TRAAK, TWIK, TASK-4 and TREK-1. For TRAAK we could specifically show that in the absence of the cysteine, the cap is stabilized by residues which are at sites homologous to that relevant in TASK-1. Thus, we propose that the conserved cysteine residues do not play a major role in the stabilization of the cap structure of homomeric K_2P_ channels and that the structures of all K_2P_ channels are likely to be stabilized by hydrophobic residues at the inner leaflet of the cap.

It is noteworthy that previous studies also found evidence that the disulfide-bridges in the cap structure are not required for the homodimerization of TWIK-2 9 and TREK-1 8 or functional expression of TASK-2 10 homodimers. Our data support this evidence for TREK-1 and additionally show that disulfide-bridges within the cap structures are also not relevant for dimerization or function of other K_2P_ channels like TWIK-1, TASK-4 and TRAAK. Thus, the question arises why do K_2P_ channels harbor a cysteine at the tip of the cap structure, when it is on the other hand not relevant for the stability of the cap and the functional expression of the homomeric channels. Here, one explanation might be that the cysteine is required for hetero dimerization with other K_2P_ channels that have ‘non-matching’ lipophilic residues at the inner leaflets of the cap, enabling also a hetero dimerization with more distantly related K_2P_ channels. In fact it has been described that heterodimers might be formed via disulfide-bridges between TWIK-1 and the TREK-1, TREK-2 and TRAAK channels that belong into another K_2P_ channel subfamily^8^. However, this idea that the cysteine is predominantly important for hetero dimerization with other K_2P_ channel subfamilies is also challenged by two studies (1) Plant *et al*. showed the lack of FRET signals for TWIK-1 with TREK-1, which appears to be in direct contradiction to the hetero dimerization postulated by Hwang *et al*. (2) In addition, it was reported that the cysteine containing channel TWIK-1 can assemble with TASK-1 or TASK-3 17 which lack a cysteine in the cap structure, thus hetero dimerization with more distantly related K_2P_ channels does not necessarily require a cysteine in the cap domain.

Our study reveals that disulfide-bridges at the tip of the cap structures of K_2P_ channels are not essential for channel assembly and functional expression, but rather seem to stabilize the cap in a way that hydrophobic residues at the inner leaflets of the cap can interact with each other. Consistently, our data indicate that the formation of cysteine-free cap structures must be conserved to that of K_2P_ channels containing an extracellular disulfide-bridge.

## Methods

### Sequence alignment and coiled-coil prediction

For sequence alignments of the extracellular M1-P1 linkers of different human K_2P_ channels the following accession numbers were used: TASK-1, NM_002246; TASK-3, AF212829; TASK-5, NM_022358; TASK-2, NM_003740; TASK-4, NM_031460; TALK-1a, NM_032115; TREK-1b, AF004711; TREK-2c, NM_138317; TRAAK, AF247042; TWIK-1, NM_002245; TWIK-2, NM_004823; THIK-1, NM_022054; THIK-2, NM_022055; TRESK, NM_181840. For coiled-coil predictions in the extracellular M1-P1 linkers the Network Protein Sequence (NPS) Analysis tool was used^11^.

### Site-directed mutagenesis

Mutations in the human TASK-1 (*KCNK3*, NM_002246), TRAAK (*KCNK4*, AF247042), TWIK-1 (*KCNK1*, U33632), TASK-3 (*KCNK9*, AF212829), TREK-1b (*KCNK2*, AF004711) or TASK-4 (*KCNK17*, AF358910) cDNA were introduced with the QuickChange Site Directed Mutagenesis Kit (Stratagene) according to the instructions of the manufacturer.

### Oocyte preparation, cRNA synthesis and cRNA injection

Oocytes were obtained from anesthetized *Xenopus laevis* frogs and incubated in OR2 solution containing in mM: NaCl 82.5, KCl 2, MgCl_2_ 1, HEPES 5 (pH 7.5) substituted with 2 mg/ml collagenase II (Sigma) to remove residual connective tissue. Subsequently, oocytes were stored at 18 °C in ND96 solution containing in mM: NaCl 96, KCl 2, CaCl_2_ 1.8, MgCl_2_ 1, HEPES 5 (pH 7.5), supplemented with 33.6 μM gentamycine, 2.5 mM sodium pyruvate and 0.5 mM theophylline. All channels were subcloned into an oocyte expression vector. Subsequently, cDNA was linearized and cRNA was synthesized with mMESSAGE mMACHINE-Kit (Ambion). The quality of cRNA was tested using gel electrophoresis. cRNA was quantified using a UV-Vis spectrophotometer (NanoDrop 2000). Oocytes were each injected with 50 nl of cRNA. For the functional expression studies and for the chemiluminecence assay of the different TASK-1 and TRAAK constructs 5 ng of cRNA were injected per oocyte. For TWIK-1, TREK-1 and TASK-4 recordings 20 ng, 1.25 ng and 25 ng of cRNA were injected, respectively. For co-expression studies 0.025 ng TASK-3 was co-injected with 2.5 ng of TASK-1 cRNA per oocyte. TASK-3 is known as one of the best expressing K_2P_ channel, while TASK-1 is due to binding to p11 primarily retained in the ER^13^,^18^. As TASK-3 has in the oocyte expression system a much more pronounced expression than TASK-1 18, we performed TEVC experiments to find the amounts of cRNA that need to be injected to get a similar expression. Here, we found that TASK-1 and TASK-3 generate similar current amplitudes, when the oocytes were injected with 0.025 ng of TASK-3 and 2.5 ng of TASK-1. Therefore, these concentrations were used for our co-expression studies.

### TEVC recordings in *Xenopus laevis* oocytes

All TEVC recordings were performed 48 h after cRNA injection at room temperature (20–22 °C) with a TurboTEC 10CD (npi) amplifier and a Digidata 1200 Series (Axon Instruments) as A/D converter. Micropipettes were made from borosilicate glass capillaries GB 150TF-8P (Science Products) and pulled with a DMZ-Universal Puller (Zeitz). Recording pipettes had a resistance of 0.5–1.5 MΩ and were filled with 3 M KCl solution. As recording solution ND96 was used. Current was analyzed with voltage steps from a holding potential of –80 mV. A first test pulse to 0 mV of 1 s duration was followed by a repolarizing step to –80 mV for 1 s, directly followed by another 1 s test pulse to +40 mV. The sweep time interval was 10 s. Data were acquired with Clampex 10 (Molecular Devices) and analyzed with Clampfit 10 (Molecular Devices) and Origin 7 (OriginLab Corporation).

### Chemiluminescence assay in *Xenopus* oocytes

Surface expression of TASK-1 channel constructs was studied using a chemiluminescence based assay. A hemagglutinin (HA)-epitope (YPYDVPDYA), preceded and followed by a proline–glycine–glycine linker, was inserted in the extracellular P2-M4 loop of human TASK-Δi20 at amino acid position 214. cRNA of HA-tagged channels was injected into *Xenopus* oocytes and surface expression was analyzed after 48 h. First, oocytes were incubated in ND96 plus 1% BSA on ice for 30 min to reduce unspecific antibody binding, followed by incubation with primary antibody (rat anti-HA (Roche), 1:100) for 1 h, extensive washing and incubation with secondary antibody (goat anti-rat-IgG, HRP-coupled (Dianova), 1:500) for 1 h. Oocytes were again extensively washed, before they were individually placed into a vial with 20 μl of luminescence substrate (SuperSignal Femto (Thermo Scientific)). Light emission was detected with a GloMax luminometer (Promega).

### Western blot analysis

cRNA of HA-tagged wild-type or mutant TASK-1 channels was injected into *Xenopus* oocytes and after 48 h whole oocytes were lysed and protein extraction was carried out as described previously^19^. Briefly, 20 oocytes were used for each lysis reaction. The oocytes were homogenized in 400 μl lysis buffer (NaCl, 150 mM; Tris/HCl, 20 mM; Triton X-100, 1%; Protease Inhibitor Cocktail (Roche), 10 μl; pH 7.5). Insoluble material was separated by centrifugation (13000 rpm) for 15 min at 4 °C. Protein was quantified using Bradford assay. Supernatant containing similar protein amounts were mixed with SDS sample buffer in the presence or absence of 50 mM DTT as indicated, heat denatured at 95 °C for 5 minutes, separated on 10% SDS-polyacrylamide gels and visualized by immunoblotting. Primary anti-HA antibodies (Roche) were used and the binding of the primary antibodies was detected using peroxidase-conjugated anti-rat IgG (Pierce), respectively and a chemiluminescent extended-duration substrate (SuperSignal WEST Dura, Pierce). Uninjected oocytes were used as negative control. Band intensities were analyzed from four to eleven independent western blot experiments with ImageJ software (NIH), background subtracted and normalized to wild-type TASK-1 without DTT. For all western blots Bradford assays were performed to ensure loading of equal amounts of total protein. Ponceau S staining after blotting was used to verify correct protein transfer and ensure equal lane loading.

HeLa cells were transfected with human TASK-1-Δi20 pEGFP^13^, human TASK-3 pEGFP or human TWIK-1 pRAT, respectively using JetPRIME (Peqlab). After 48 h cells were lysed in the lysis buffer. After centrifugation, supernatants were mixed with SDS sample buffer in the presence or absence of 50 mM DTT as indicated, heat denatured, separated on 10% SDS-polyacrylamide gels and visualized by immunoblotting. As primary antibodies anti-GFP (ab290, abcam) or anti-TWIK-1 (kindly provided by Florian Lesage) were used and the binding of the primary antibodies was detected using peroxidase-conjugated anti-rabbit antibodies (1:2000) and a chemiluminescent extended-duration substrate (SuperSignal WEST Dura, Pierce).

### Cell culture and transfection

HeLa cells were cultivated at 37 °C and 5% CO_2_ in DMEM medium (Invitrogen) supplemented with 10% FCS and 1% Penicillin/Streptomycin solution (Invitrogen). For imaging experiments cells were grown on glass bottom (WillCo) 35 mm petri dishes, respectively. At a confluency of 60–70% cells were transfected with FuGENE6 (Promega) or JetPRIME (Peqlab). For fluorescence imaging experiments, a total amount of 1 μg of cDNA per 35 mm dish was used for transfection.

### Fluorescence imaging

Fluorescence microscopy of transfected HeLa cells was performed with an Axio Observer.Z1 microscope (Zeiss), equipped with a Zeiss Plan‐Apochromat 60 × /1.40 Oil DIC objective. During live cell imaging, cells were maintained at 37 °C using an objective heater (Bioptechs). A standard Zeiss filter set (38HE) was used. Images were taken with a Zeiss 12bit ‘AxioCam MRm’ camera, and digital images were processed using Zeiss AxioVision Software.

### Homology modeling and MD simulations

Four TASK-1 homology models were built using the following crystal structures as templates: domain-swapped TRAAK -PDB ID 4I9W-3, non domain-swapped TRAAK -PDB ID 3UM7-2, TWIK-1 -PDB ID 3UKM-4 and TREK-2 -PDB ID 4BW5-6. The TASK-1 homology models were made using ICM software^20^ and according to the multiple alignment reported by Brohawn *et al*. in 2012 2. The domain-swapped and non domain-swapped TRAAK crystal structures (PDB IDs 4I9W and 3UM7 respectively) as well as the four TASK-1 homology models were embedded into a pre-equilibrated phosphatidyl oleoyl phosphatidylcholine (POPC) bilayer in a periodic boundary condition box (15 × 15 × 15 Å^3^) with pre-equilibrated SPC water molecules. Two K^+^ ions were associated to the models at positions S2 and S4 of the selectivity filter, and two water molecules at positions S1 and S3. Finally, the systems were neutralized by adding K^+^ counter ions to balance the net charge of the systems and KCl at a concentration of 0.096 M was added to simulate physiological conditions of the channels. An excluded region for counter ions was set at 5 Å from the selectivity filter of the models. MD simulations were performed employed OPLS-AA force field^21^ within Desmond package^22^ contained in Maestro 9.2 suite. The systems were equilibrated using default relaxation protocol.

MD simulations were performed during 10 ns for all systems. For the TASK-1 homology model based on TREK-2 the model subsequently ran additional 90 ns. In all MD simulations the alpha helixes of the channels were energy-restricted with a spring constant of 0.5 kcal mol^−1^ Å^−2^ at constant temperature (300 K) and pressure (1.01325 bar) in an isothermal-isobaric ensemble. Data were collected every 4 ps during the trajectory. The stability of the models during the MD simulations was validated by calculating the Root-Mean-Square Deviation (RMSD) (Supplementary Fig. S9). The values of Table 1 and the Supplemental Tables S1–5 are an average (and its respective standard deviation) of the distances between the residues during the Molecular Dynamics Simulations (MDS). During the MDS the structures were collected each 4 ps. A total of 2500 structures were obtained during each 10 ns MDS and the distances between the residues were calculated using an in house written script in tcl language.

Interactions of key residues of the helical cap were evaluated measuring the carbon-carbon atom distances from predefined carbon atoms. The carbon atoms selected/predefined are shown in ball representation in Supplementary Fig. S3. They are the carbon atoms before the last ramification of the side chains. The distances were measured using a script in house written in tcl language. A cut-off of 6.0 Å (instead 4.0 Å) was used to probe for interactions, as the carbon atoms selected for the automatic distance measurements do not necessarily reflect the closest atom for interactions and the space of the H atoms are not considered.

### Animal experiments

All animal experiments were carried out in accordance with EU Directive 2010/63/EU for animal experiments. The work with *Xenopus* at the University of Marburg with all experimental protocols were approved by the Regierungspräsidium Gießen, Germany (V54-19c 20 15 h 02 MR 20/28 Nr.A 4/2013).

### Statistical analyses

All values, unless stated otherwise are expressed as Mean ± SEM. Significance was assessed using unpaired two tailed Student’s T-Test. Asterisks indicate significance: *p < 0.05; **p < 0.01; ***p < 0.001. The number of experiments are included in the Figure.

## Additional Information

**How to cite this article**: Goldstein, M. *et al*. Functional mutagenesis screens reveal the `cap structure´ formation in disulfide-bridge free TASK channels. *Sci. Rep.* **6**, 19492; doi: 10.1038/srep19492 (2016).

## Supplementary Material

Supplementary Information

## Figures and Tables

**Table 1:**
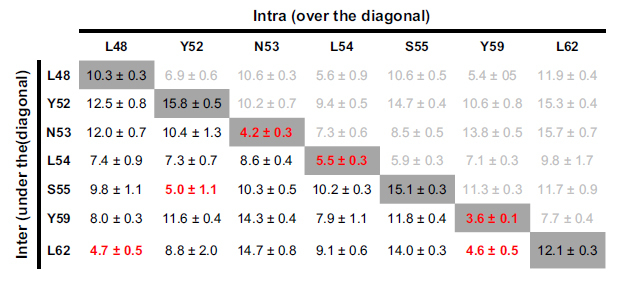
Intersubunit interactions in a TASK-1 homology model based on domain-swapped TREK-2.

The diagonal line, highlighted in gray, indicates the distance to the same residue in the neighboring subunit of the dimeric protein. Intersubunit distances are listed under the diagonal line and are written black. Intrasubunit interactions which should be less relevant for the dimerization of the channel are listed over the diagonal line and written in a light gray. Distances are measured from a predefined carbon of the amino acid side chain, lacking also the hydrogen atoms, and thus a more remote cut-off of 6 Å was used (Supplementary Fig. S3 and Methods). Intersubunit distances under 6 Å are indicated in red. Mean ± SDEV.
