# Supplementary material for: Functional mutagenesis screens reveal the ‘cap structure’ formation in disulfide-bridge free TASK channels
Source: Sci Rep. 2016 Jan 22;6:19492. doi: 10.1038/srep19492 (PMC4726246; doi:10.1038/srep19492)
Supplement: Supplementary Information [file srep19492-s1.pdf]

Supplementary Information

Functional mutagenesis screens reveal the `cap structure´ formation in disulfide-bridge free TASK channels

Matthias Goldstein[1,*], Susanne Rinné[1,*], Aytug K. Kiper[1,*], David Ramírez[2], Michael F. Netter[1], Daniel Bustos[2], Beatriz Ortiz-Bonnin[1], Wendy González[2] & Niels Decher[1,§]

[1]Institute for Physiology and Pathophysiology, University of Marburg, 35037 Marburg, Germany.

[2]Center for Bioinformatics and Molecular Simulation, University of Talca, Talca, Chile

Supplementary Contents:

Supplementary Figures S1-S9
Supplementary Tables S1-S5
Supplementary References

Supplementary Figures

a

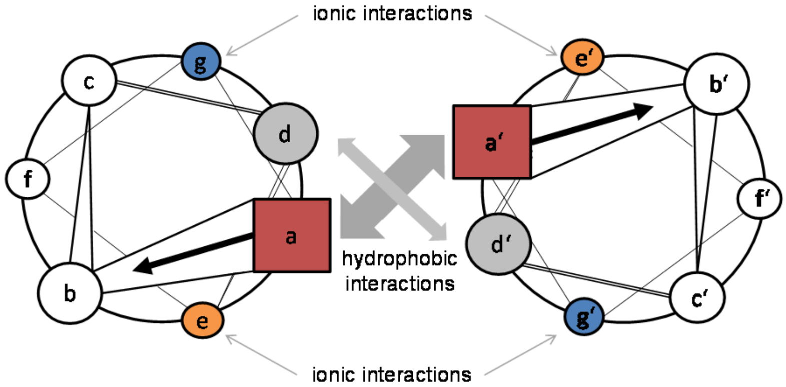

b

		repeats
TASK-1	$_{31}$SEPELIERQRLELRQQELRARYN-LSQGGYEELERVVLRLKPHKAG$_{75}$	4
TASK-3	$_{31}$SDHEMREEEKLKAEEIRIKGKYN-ISSEDYRQLELVILQSEPHRAG$_{75}$	4
TASK-5	$_{31}$SEAESGRQRLLVQKRGALRRKFG-FSAEDYRELERLALQAEPHRAG$_{75}$	2
TASK-2	$_{30}$EPHWKEAKKNYYTQKLHLLKEFPCLGQEGLDKILEVVSDAAGQGVA$_{73}$	-
TASK-4	$_{47}$GRAAQDSSRSFQRDKWELLQNFTCLDRPALDSLIRDVVQAYKNGAS$_{90}$	-
TALK-1	$_{39}$RQAEAQSRDQFQLEKLRFLENYTCLDQWAMEQFVQVIMEAWVKGVN$_{82}$	-
TREK-1	$_{87}$QPHEISQRTTIVIQKQTFISQHSCVNSTELDELIQQIVAAINAGII$_{130}$	2
TREK-2	$_{100}$QPFESSQKNTIALEKAEFLRDHVCVSPQELETLIQHALDADNAGVS$_{145}$	-
TRAAK	$_{55}$QPHEQQAQRELGEVREKFLRAHPCVSDQELGLLIKEVADALGGAD$_{100}$	2
TWIK-1	$_{48}$LPYEDLLRQELRKLKRRFLEEHECLSEQQLEQFLGRVLEASNYGVS$_{91}$	3
TWIK-2	$_{32}$GPHEARLRAELETLRAQLLQRSPCVAAPALDAFVERVLAAGRLGRV$_{75}$	2
THIK-1	$_{43}$LAHERQAKQRWEERLANFSRGHN-LSRDELRGFLRHYEEATRAGIR$_{87}$	2
THIK-2	$_{62}$SPGEAEARARWGATLRNFSAAHG-VAEPELRAFLRHYEAALAAGVR$_{106}$	-
TRESK	$_{48}$QVLVAADDGEFEKFLEELCRILNCSETVVEDRKQDLQGHLQKVKPQ$_{93}$	2

Supplementary Fig. S1 I Coiled-coil prediction in the M1-P1 linker of K_{2P} channels without a cysteine in the extracellular cap

(**a**) The coiled-coil is a common structural motif, consisting of two to five α-helices wrapped around each other into a left-handed helix forming a supercoil (Mason et al., 2004). Coiled-coil structures usually contain a repeated pattern of hydrophobic (h) and charged (c) amino-acid residues. \`hxxhcxc´ refers to a heptad repeat re-occuring after every two turns of the helix. The positions within the heptad repeat are commonly labeled as 'a-b-c-d-e-f-g', where 'a' (*red*) and 'd' (*gray*) are the hydrophobic core positions, often occupied by the amino acids isoleucine, leucine, or valine, thus stabilizing helix dimerization through hydrophobic and van-der-Waals interactions, whereas 'e' (*orange*) and 'g' (*blue*) are typically solvent-exposed, polar residues (e.g. glutamate or lysine) that give specificity between the two helices through interhelical electrostatic interactions (Mason et al., 2004). The remaining three positions ('b', 'c', and 'f', *white*) must be all hydrophilic, as these will form helical solvent exposed surfaces (O´Shea et al. 1993; Graddis et al., 1993). (**b**) For coiled-coil predictions in the extracellular M1-P1 linkers of K_{2P} channels the Network Protein Sequence (NPS) analysis tool was used (Combet et al., 2000). The number of heptad repeats are listed beside the alignment. Only for TASK-1 (p=0.991) and TASK-3 (p=0.542) a 4-fold heptad repeat prediction was observed.

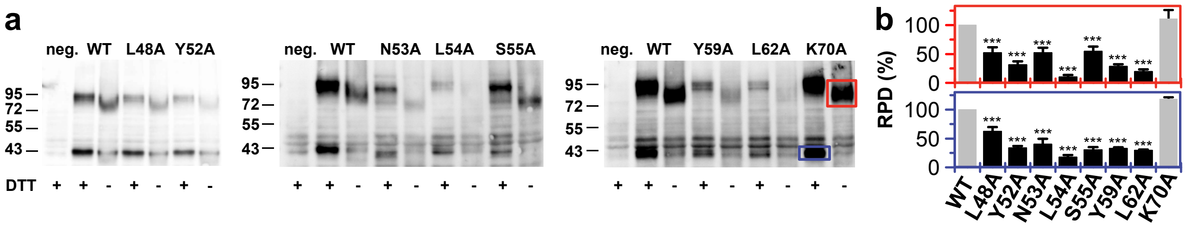

Supplementary Fig. S2 I Protein expression analysis under reducing and non-reducing conditions

(**a**) Oocytes injected with indicated TASK-1-HA constructs were lysed and denatured under reducing (+DTT, 50 mM) or non-reducing (-DTT) conditions. Similar protein amounts were separated on SDS gels, blotted and stained with anti-HA antibodies and peroxidase labelled secondary antibodies. Protein sizes are given in kD (kilo Dalton). As a negative control (neg.) protein lysates of non-injected oocytes were used. The blue box is an example indicating the band for monomeric and the red box for dimeric TASK-1, as here the quantification of pixel intensities for (**b**) were performed. Note that there are two unspecific bands located above the monomeric protein. (**b**) Analysis of the pixel intensities of the protein signals corresponding to monomeric TASK-1 (+DTT) normalized to TASK-1 wild-type with DTT (lower panel) or of the protein signals corresponding to the dimeric TASK-1 (-DTT) (upper panel) normalized to TASK-1 wild-type without DTT. Image J was used for pixel intensity analysis. RPD: relative pixel density.

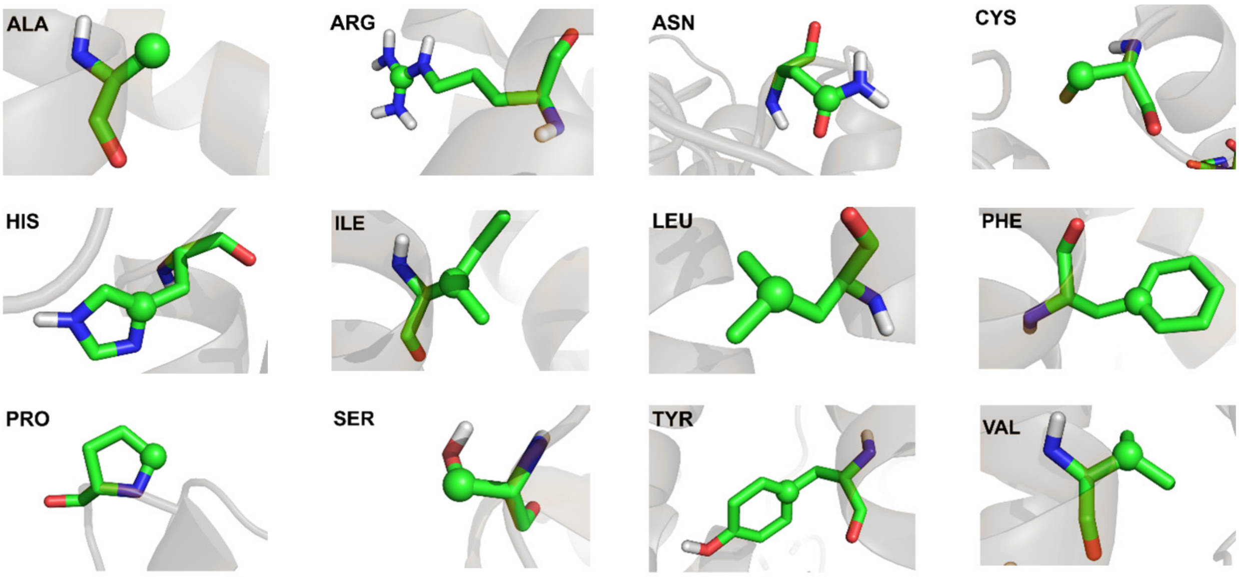

Supplementary Fig. S3 I Predefined C-atoms used for the distance measurements during the MD simulations

Key residues of the helical cap are depicted in stick mode. The C-atoms which were considered for the distance measurements during the MD simulations are indicated in ball representation (see Methods).

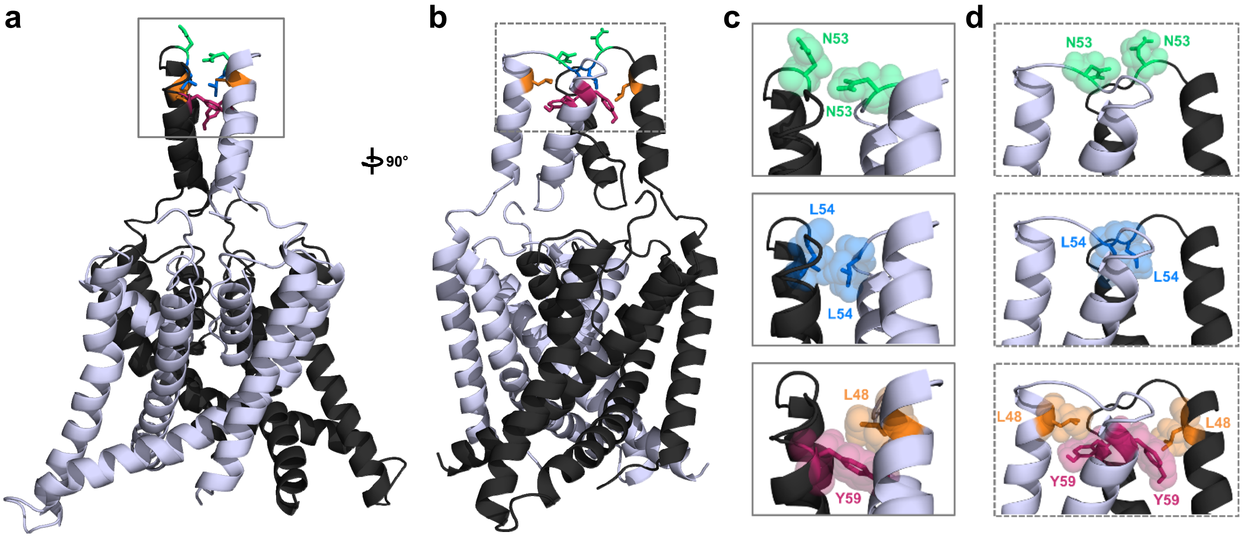

Supplementary Fig. S4 I TASK-1 cap structure model based on the non domain-swapped TRAAK (PDB ID 3UM7).

TASK-1 cap structure illustrated after 10 ns of MD simulations. The two different subunits are shown in *gray* and *light gray*. TASK-1 cap model with the residues illustrated that were identified as essential for channel expression and are involved in intersubunit interactions during the 10 ns MD simulations (Supplementary Table S2). Note that only L48, N53, L54 and Y59 are involved in intersubunit interactions in this model. (**a**) Complete structure of the TASK-1 model, and (**b**) after rotating by 90°. (**c-d**) Close-up in the cap on specific pairs of amino acids involved in intersubunit interactions. (**c**) Zoom-in to the boxed area of (**a**) illustrating interacting residues, and (**d**) after Zoom-in to the boxed area of (**b**).

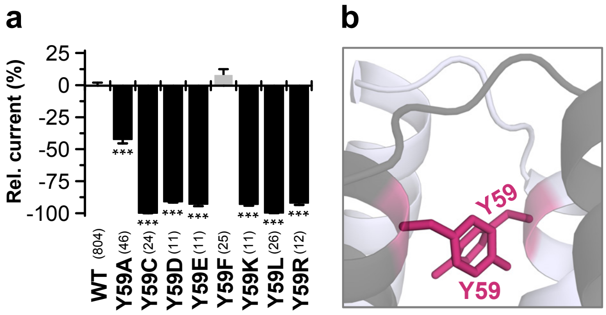

Supplementary Fig. S5 I The aromatic moiety of Y59 is functional relevant and not H-bonds to the hydroxyl group

(**a**) Relative current density of Y59 mutations analyzed at +40 mV. ***, p<0.001. Unpaired Student´s T-Test. Significance was probed against wild-type TASK-1 (WT). Mean ± SEM. The number of experiments are included above the construct name. (**b**) Position of Y59 using a TASK-1 homology model based on domain-swapped TREK-2 after 100 ns MD simulation.

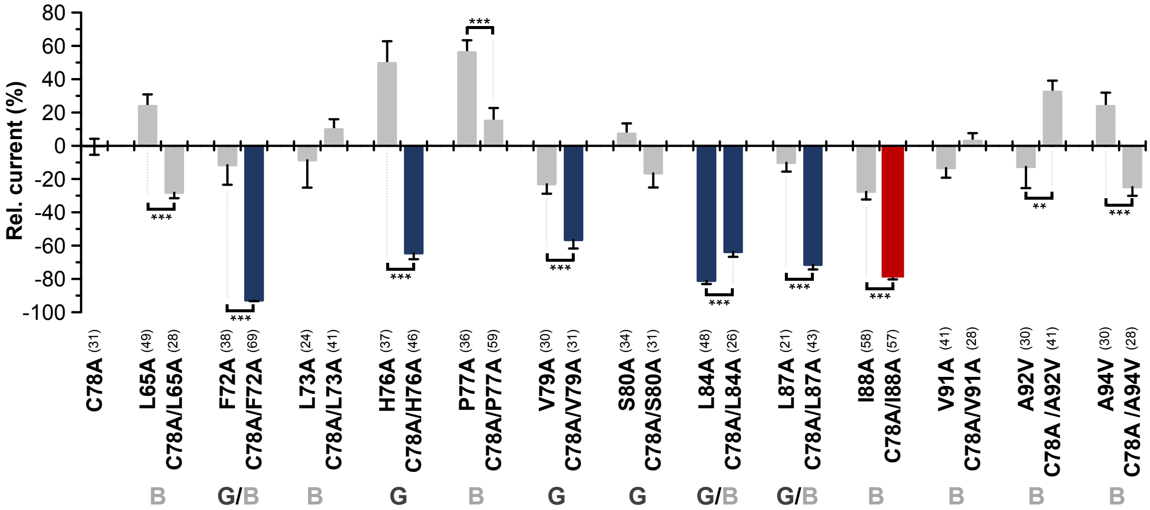

Supplementary Fig. S6 I Analysis of mutations introduced in TRAAK at sites homologues to the TASK-1 \`hits´

The current amplitudes were studied for the mutations alone or as double mutations with C78A. \`G´ in black indicates homologous TRAAK sites according to the residues we identified in the current study as functional relevant in TASK-1. Here F72 in TRAAK corresponds to L48 in TASK-1, H76 to Y52, C78 to N53, V79 to L54, S80 to S55, L84 to Y59 and L87 to L62. \`B´ in grey indicates residues that were proposed by MacKinnon´s group to be functional relevant within the cap of the TRAAK crystal structure (Brohawn et al., 2012). The red bar for C78A/I88A indicates that I88 was the only functional relevant site proposed by Brohawan et al., that was not homologous to the residues we identified in TASK-1. **, $p<0.01$; ***, $p<0.001$. Unpaired Student´s T-Test. Significance of the double mutation was probed against the respective single mutation without C78A. Mean ± SEM. The number of experiments are provided in parenthesis above the name of the respective construct.

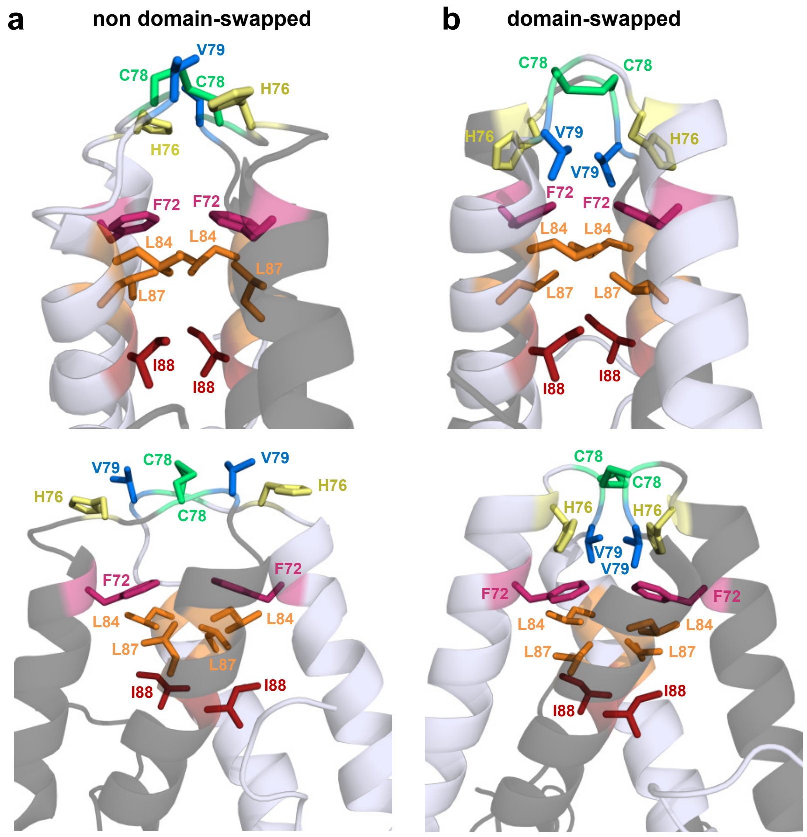

Supplementary Fig. S7 I Functional relevant TRAAK residues in the non domain-swapped and domain-swapped crystal structures

The two different subunits are shown in *gray* and *light gray*. (**a**) The non domain-swapped crystal structure (PDB ID 3UM7) illustrated in the same orientation as in Brohawn et al., 2012 (upper panel) and below in an alternative orientation. (**b**) The domain swapped crystal structure (PDB ID 4I9W) illustrated in the same orientation as in Brohawn et al., 2012 (upper panel) and below in an alternative orientation.

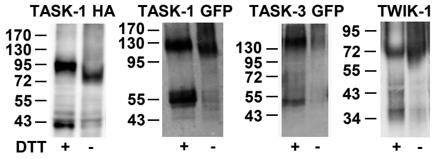

Supplementary Fig. S8 I Protein expression analysis under reducing and non-reducing conditions

Oocytes injected with TASK-1 HA cRNA were lysed and denatured under reducing (+DTT, 50 mM) or non-reducing (-DTT) conditions. HeLa cells were transfected with TASK-1 pEGFP, TASK-3 pEGFP or TWIK-1 pRAT, respectively. Cells were lysed and denatured under reducing (+DTT) and non-reducing (-DTT) conditions and western blot analysis were performed using anti-HA, anti-GFP or anti-TWIK-1 antibodies, respectively and corresponding secondary peroxidase conjugated antibodies.

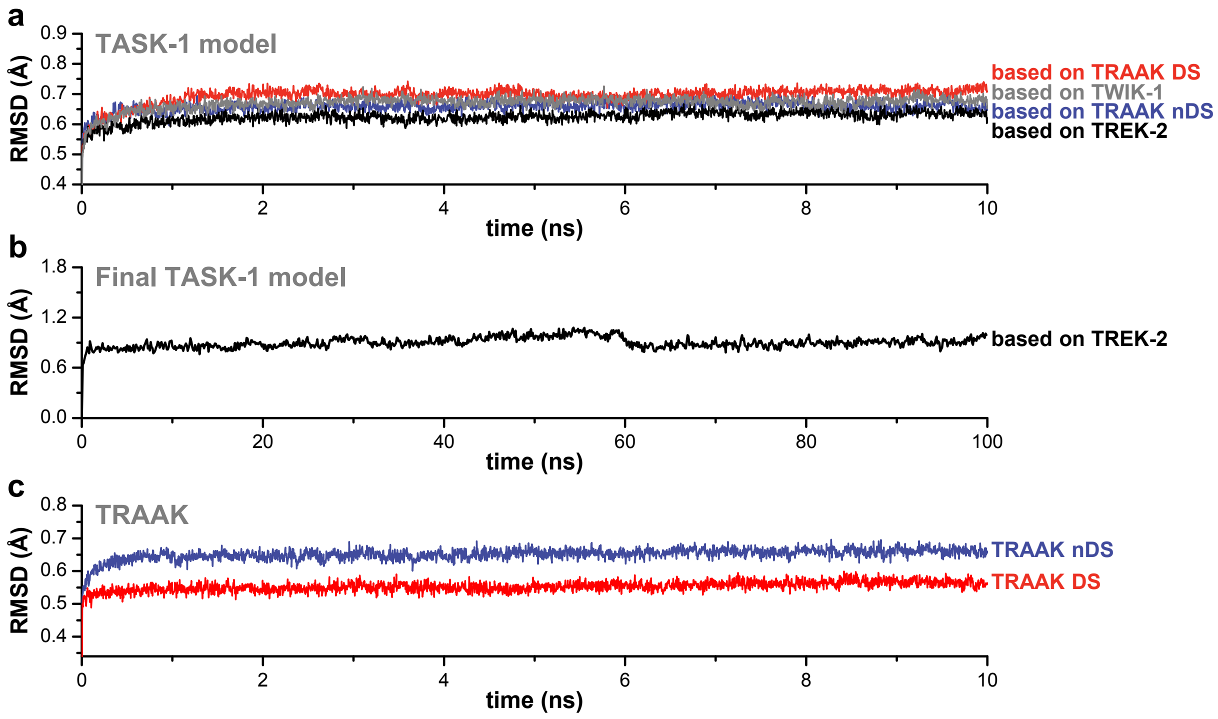

Supplementary Fig. S9 I Root-mean-square deviation (RMSD) values during the MD simulations

Time dependence of the RMSD for the backbones from starting structures during the MD simulation. DS, domain-swapped; nDS, non domain-swapped. (**a**) RMSDs for the TASK-1 homology models during the 10 ns MDs. TRAAK DS, PDB ID 4I9W; TRAAK nDS, PDB ID 3UM7; TWIK-1, PDB ID 3UKM and TREK-2, PDB ID 4BW5. (**b**) RMSD of the TASK-1 model based on TREK-2 (4BW5) during 100 ns of MD simulations. (**c**) TRAAK crystal structures during the 10 ns MDs. TRAAK DS, PDB ID 4I9W and TRAAK nDS, PDB ID 3UM7.

Supplementary Tables

	Intra (over the diagonal)						
**Inter (below the diagonal)**	**L48**	**Y52**	**N53**	**L54**	**S55**	**Y59**	**L62**
**L48**	16.6 ± 1.1	13.2 ± 0.6	4.8 ± 0.5	10.3 ± 0.9	11.7 ± 0.5	9.9 ± 0.7	4.9 ± 0.4
**Y52**	19.5 ± 1.5	12.9 ± 1.7	10.5 ± 0.8	7.1 ± 1.6	11.0 ± 2.4	11.9 ± 1.7	15.5 ± 0.8
**N53**	15.3 ± 0.9	16.9 ± 1.2	14.5 ± 0.8	6.8 ± 0.3	7.0 ± 0.4	7.1 ± 0.5	6.4 ± 0.8
**L54**	13.4 ± 0.9	10.9 ± 1.2	11.7 ± 1.0	6.8 ± 0.9	6.7 ± 0.5	5.5 ± 0.7	10.8 ± 0.9
**S55**	15.3 ± 0.8	15.4 ± 1.2	15.5 ± 0.6	12.5 ± 0.9	18.3 ± 0.4	7.3 ± 0.6	11.7 ± 0.5
**Y59**	8.8 ± 0.8	11.9 ± 0.8	8.5 ± 0.6	7.2 ± 1.0	11.9 ± 0.4	**5.8 ± 0.5**	7.9 ± 0.5
**L62**	12.7 ± 0.8	18.6 ± 1.2	12.4 ± 0.7	12.5 ± 0.8	14.0 ± 0.3	7.4 ± 0.7	8.4 ± 0.5

Supplementary Table S1 I Intersubunit interactions in a TASK-1 homology model based on TWIK-1 during 10 ns of MD simulations

The diagonal line, highlighted in gray, indicates the distance to the same residue in the neighboring subunit of the dimeric protein. Intersubunit distances are listed under the diagonal line and are written in black. Intrasubunit interactions which should be less relevant for the dimerization of the channel are listed over the diagonal line and written in a light grey. Distances are measured from a predefined carbon of the amino acid side chain, lacking also the hydrogen atoms, and thus a more remote cut-off of 6 Å was used (Supplementary Fig. S3 and Methods). Intersubunit distances under 6 Å are indicated in red. One putative intersubunit interaction was identified. Mean ± SDEV.

	Intra (over the diagonal)						
**Inter (below the diagonal)**	**L48**	**Y52**	**N53**	**L54**	**S55**	**Y59**	**L62**
**L48**	12.0 ± 0.5	10.0 ± 1.2	7.5 ± 1.7	5.5 ± 0.6	10.7 ± 1.0	12.2 ± 0.8	6.4 ± 0.7
**Y52**	14.1 ± 1.0	13.1 ± 1.0	5.6 ± 1.3	9.8 ± 0.5	7.0 ± 1.7	16.4 ± 1.0	13.5 ± 2.5
**N53**	10.5 ± 0.7	9.4 ± 0.7	**5.4 ± 0.7**	6.6 ± 0.7	7.3 ± 0.5	13.3 ± 0.6	11.0 ± 1.7
**L54**	7.7 ± 1.3	12.9 ± 0.6	8.0 ± 1.0	**5.4 ± 0.3**	6.6 ± 0.4	8.2 ± 0.8	5.5 ± 0.5
**S55**	9.0 ± 0.8	13.4 ± 0.8	8.0 ± 1.3	9.8 ± 0.6	13.3 ± 0.6	10.7 ± 0.3	9.9 ± 1.5
**Y59**	**4.9 ± 0.4**	15.3 ± 1.7	11.5 ± 2.0	7.3 ± 1.2	13.4 ± 1.6	10.9 ± 0.6	7.8 ± 1.4
**L62**	10.0 ± 0.7	16.6 ± 1.1	12.3 ± 1.2	7.9 ± 1.8	12.6 ± 0.6	16.6 ± 1.1	7.6 ± 0.4

Supplementary Table S2 I Intersubunit interactions in a TASK-1 homology model based on non-domain swapped TRAAK during 10 ns of MD simulations

The diagonal line, highlighted in gray, indicates the distance to the same residue in the neighboring subunit of the dimeric protein. Intersubunit distances are listed under the diagonal line and are written in black. Intrasubunit interactions which should be less relevant for the dimerization of the channel are listed over the diagonal line and written in a light grey. Distances are measured from a predefined carbon of the amino acid side chain, lacking also the hydrogen atoms, and thus a more remote cut-off of 6 Å was used (Supplementary Fig. S3 and Methods). Intersubunit distances under 6 Å are indicated in red. Three putative intersubunit interactions were identified. Mean ± SDEV.

Inter (below the diagonal) \ Intra (over the diagonal)	L48	Y52	N53	L54	S55	Y59	L62
**L48**	8.1 ± 0.2	7.4 ± 0.6	9.7 ± 0.5	5.6 ± 0.3	10.3 ± 0.3	4.8 ± 0.9	7.9 ± 1.5
**Y52**	10.2 ± 0.6	13.4 ± 0.6	10.0 ± 1.1	9.7 ± 0.8	15.0 ± 0.3	11.8 ± 0.9	12.9 ± 1.3
**N53**	11.4 ± 1.4	8.2 ± 1.2	7.0 ± 0.8	7.3 ± 0.3	8.3 ± 0.8	13.6 ± 0.7	13.2 ± 0.8
**L54**	6.1 ± 0.5	**5.0 ± 0.5**	9.2 ± 2.5	6.5 ± 0.4	6.1 ± 0.6	7.5 ± 0.4	6.7 ± 0.5
**S55**	11.2 ± 0.5	**5.3 ± 0.8**	13.0 ± 2.0	12.3 ± 0.8	18.8 ± 0.3	11.0 ± 0.3	10.0 ± 0.9
**Y59**	9.1 ± 1.1	11.5 ± 1.1	15.4 ± 2.4	9.6 ± 1.0	14.8 ± 1.1	8.7 ± 0.5	6.5 ± 1.4
**L62**	**5.1 ± 0.5**	8.0 ± 1.9	13.6 ± 1.3	9.0 ± 0.9	13.6 ± 0.3	6.1 ± 1.8	8.1 ± 0.4

Supplementary Table S3 I Intersubunit interactions in a TASK-1 homology model based on domain-swapped TRAAK during 10 ns of MD simulations

The diagonal line, highlighted in gray, indicates the distance to the same residue in the neighboring subunit of the dimeric protein. Intersubunit distances are listed under the diagonal line and are written in black. Intrasubunit interactions which should be less relevant for the dimerization of the channel are listed over the diagonal line and written in a light grey. Distances are measured from a predefined carbon of the amino acid side chain, lacking also the hydrogen atoms, and thus a more remote cut-off of 6 Å was used (Supplementary Fig. S3 and Methods). Intersubunit distances under 6 Å are indicated in red. Three putative intersubunit interactions were identified. Mean ± SDEV.

Intra (over the diagonal)

Inter (under the diagonal)	L65	V68	F72	L73	H76	P77	C78	V79	S80	L84	L87	I88	V91	A92	A94
**L65**	15.4 ± 0.3	5.5 ± 0.3	11.2 ± 0.3	12.1 ± 0.3	18.0 ± 0.5	15.0 ± 0.5	19.9 ± 0.7	22.8 ± 0.3	19.8 ± 0.5	15.1 ± 0.4	9.4 ± 0.3	11.8 ± 0.3	5.9 ± 0.3	9.9 ± 0.3	4.5 ± 0.2
**V68**	16.0 ± 0.3	17.0 ± 0.3	7.8 ± 0.3	10.5 ± 0.5	14.9 ± 0.6	11.1 ± 0.6	16.9 ± 0.8	19.7 ± 0.3	15.9 ± 0.5	13.1 ± 0.3	6.9 ± 0.3	10.7 ± 0.3	6.2 ± 0.4	10.4 ± 0.4	6.1 ± 0.2
**F72**	15.9 ± 0.6	14.9 ± 0.5	9.3 ± 0.2	6.3 ± 1.0	8.3 ± 1.0	4.3 ± 0.3	9.2 ± 0.4	12.5 ± 0.2	10.1 ± 0.9	8.9 ± 0.7	4.9 ± 0.9	10.8 ± 0.9	9.6 ± 0.7	14.4 ± 0.8	13.2 ± 0.4
**L73**	20.6 ± 0.5	18.6 ± 0.4	13.4 ± 0.7	18.5 ± 0.5	7.0 ± 0.5	8.7 ± 0.8	11.9 ± 0.7	16.3 ± 0.5	15.5 ± 0.6	14.7 ± 0.5	10.5 ± 0.3	16.3 ± 0.4	13.4 ± 0.4	18.8 ± 0.3	15.6 ± 0.4
**H76**	23.3 ± 0.6	21.1 ± 0.6	13.9 ± 0.6	17.2 ± 0.5	14.3 ± 0.4	6.9 ± 0.1	7.6 ± 0.4	12.2 ± 0.4	12.6 ± 0.5	15.1 ± 0.6	13.1 ± 0.5	18.6 ± 0.4	17.7 ± 0.5	22.6 ± 0.4	20.7 ± 0.5
**P77**	17.6 ± 0.3	16.6 ± 0.4	9.9 ± 0.5	12.3 ± 0.6	12.0 ± 0.9	10.0 ± 0.8	6.4 ± 0.1	9.5 ± 0.3	7.1 ± 0.8	9.2 ± 0.4	7.5 ± 0.3	12.5 ± 0.3	12.8 ± 0.3	16.9 ± 0.3	16.5 ± 0.6
**C78**	19.5 ± 0.9	17.1 ± 0.7	9.2 ± 0.7	10.6 ± 0.5	7.0 ± 0.6	7.6 ± 1.0	**3.5 ± 0.2**	4.8 ± 0.2	7.0 ± 0.2	10.5 ± 0.9	12.0 ± 0.7	15.8 ± 0.7	17.3 ± 0.7	21.2 ± 0.7	21.9 ± 0.8
**V79**	19.2 ± 0.4	17.1 ± 0.4	9.8 ± 0.7	8.1 ± 0.5	**4.6 ± 0.3**	9.1 ± 1.0	7.2 ± 0.4	11.7 ± 0.4	5.6 ± 0.1	10.3 ± 0.5	13.9 ± 0.3	16.2 ± 0.3	19.2 ± 0.3	22.1 ± 0.3	24.2 ± 0.3
**S80**	16.6 ± 0.4	16.1 ± 0.4	9.8 ± 0.8	7.8 ± 0.3	9.0 ± 0.6	10.9 ± 1.0	9.8 ± 0.7	13.6 ± 0.7	15.2 ± 0.5	7.6 ± 0.4	10.5 ± 0.5	12.5 ± 0.4	15.6 ± 0.4	18.0 ± 0.4	21.3 ± 0.4
**L84**	9.3 ± 0.4	8.8 ± 0.4	**4.9 ± 0.3**	**5.7 ± 0.4**	10.4 ± 0.7	8.5 ± 0.6	11.6 ± 0.8	15.1 ± 0.5	13.8 ± 0.6	10.3 ± 0.4	6.2 ± 0.3	6.0 ± 0.4	10.0 ± 0.3	12.1 ± 0.4	15.7 ± 0.3
**L87**	11.3 ± 0.4	11.5 ± 0.3	8.3 ± 0.3	11.8 ± 0.4	14.8 ± 0.7	10.8 ± 0.5	12.5 ± 1.0	14.1 ± 0.5	12.8 ± 0.4	6.5 ± 0.4	6.1 ± 0.3	5.9 ± 0.2	5.5 ± 0.2	9.6 ± 0.2	10.3 ± 0.3
**I88**	6.1 ± 0.3	8.7 ± 0.3	9.5 ± 0.3	11.0 ± 0.5	16.4 ± 0.8	13.3 ± 0.5	16.6 ± 1.1	19.2 ± 0.5	17.2 ± 0.4	11.4 ± 0.4	7.5 ± 0.4	9.5 ± 0.3	5.9 ± 0.2	6.2 ± 0.2	11.1 ± 0.2
**V91**	10.1 ± 0.3	11.6 ± 0.3	12.0 ± 0.4	15.6 ± 0.4	19.5 ± 0.7	14.8 ± 0.4	17.5 ± 1.0	18.6 ± 0.5	16.3 ± 0.4	9.0 ± 0.4	7.9 ± 0.4	**5.7 ± 0.2**	**5.8 ± 0.2**	5.4 ± 0.1	5.8 ± 0.2
**A92**	8.6 ± 0.3	12.6 ± 0.4	15.1 ± 0.3	17.2 ± 0.6	22.5 ± 0.8	18.6 ± 0.5	21.8 ± 1.1	23.6 ± 0.5	21.3 ± 0.4	14.2 ± 0.5	11.6 ± 0.5	10.3 ± 0.4	7.5 ± 0.4	8.7 ± 0.3	7.3 ± 0.1
**A94**	14.9 ± 0.3	17.1 ± 0.3	17.7 ± 0.5	21.3 ± 0.4	25.0 ± 0.6	20.1 ± 0.4	22.1 ± 0.8	22.3 ± 0.4	20.3 ± 0.3	12.7 ± 0.4	13.4 ± 0.4	9.3 ± 0.4	10.8 ± 0.4	9.7 ± 0.3	14.9 ± 0.3

Supplementary Table S4 I Intersubunit interactions in the non domain-swapped TRAAK during 10 ns of MD simulations

The diagonal line, highlighted in gray, indicates the distance to the same residue in the neighboring subunit of the dimeric protein. Intersubunit distances are listed under the diagonal line and are written in black. Intrasubunit interactions which should be less relevant for the dimerization of the channel are listed over the diagonal line and written in a light grey. Distances are measured from a predefined carbon of the amino acid side chain, lacking also the hydrogen atoms, and thus a more remote cut-off of 6 Å was used (Supplementary Fig. S3 and Methods). Intersubunit distances under 6 Å are indicated in red. Six putative intersubunit interactions were identified. Mean ± SDEV.

Inter (under the diagonal)	Intra (over the diagonal)														
	L65	V68	F72	L73	H76	P77	C78	V79	S80	L84	L87	I88	V91	A92	A94
L65	14.9 ± 0.3	6.0 ± 0.4	11.2 ± 0.3	11.7 ± 0.3	16.1 ± 0.3	20.7 ± 0.3	20.8 ± 0.3	14.9 ± 0.3	16.3 ± 0.3	9.1 ± 0.4	11.0 ± 0.3	5.9 ± 0.2	9.7 ± 0.3	8.4 ± 0.4	14.7 ± 0.3
V68	15.5 ± 0.2	16.5 ± 0.3	6.8 ± 0.5	9.6 ± 0.2	11.8 ± 0.3	17.0 ± 0.3	17.6 ± 0.3	12.8 ± 0.3	15.9 ± 0.3	8.3 ± 0.3	11.2 ± 0.4	8.7 ± 0.5	11.5 ± 0.3	12.8 ± 0.6	17.0 ± 0.4
F72	15.3 ± 0.2	14.6 ± 0.5	10.2 ± 0.5	6.5 ± 0.6	5.2 ± 0.4	10.9 ± 0.5	10.3 ± 0.6	7.1 ± 1.1	11.8 ± 1.0	5.7 ± 0.6	8.8 ± 0.7	10.0 ± 0.4	12.0 ± 0.2	15.3 ± 0.3	17.6 ± 0.3
L73	19.2 ± 0.3	17.0 ± 0.3	11.9 ± 0.6	15.6 ± 0.4	8.0 ± 0.3	9.7 ± 0.3	10.3 ± 0.2	5.7 ± 0.3	7.1 ± 0.3	4.9 ± 0.2	10.6 ± 0.3	10.4 ± 0.3	14.6 ± 0.3	16.6 ± 0.4	20.4 ± 0.3
H76	17.6 ± 0.4	15.9 ± 0.4	10.1 ± 1.2	10.2 ± 0.3	7.9 ± 0.2	6.7 ± 0.1	7.1 ± 0.2	5.8 ± 0.3	11.3 ± 0.3	8.6 ± 0.5	10.9 ± 0.4	13.9 ± 0.5	15.2 ± 0.5	19.3 ± 0.6	20.3 ± 0.4
P77	23.8 ± 0.3	21.0 ± 0.3	14.4 ± 0.9	15.1 ± 0.4	10.0 ± 0.3	9.6 ± 0.6	5.3 ± 0.4	7.7 ± 0.1	10.5 ± 0.4	14.9 ± 0.3	14.6 ± 0.4	18.6 ± 0.3	20.9 ± 0.3	24.5 ± 0.3	26.0 ± 0.3
C78	20.3 ± 0.3	17.1 ± 0.3	11.1 ± 0.9	11.5 ± 0.3	**5.3 ± 0.2**	**5.4 ± 0.3**	**3.9 ± 0.1**	5.5 ± 0.1	8.5 ± 0.2	11.3 ± 0.3	13.0 ± 0.4	16.7 ± 0.3	18.3 ± 0.2	22.2 ± 0.2	22.8 ± 0.3
V79	17.4 ± 0.3	14.0 ± 0.3	7.7 ± 0.7	11.2 ± 0.3	**4.6 ± 0.2**	9.7 ± 0.3	7.2 ± 0.2	6.9 ± 0.2	5.8 ± 0.1	5.9 ± 0.2	8.7 ± 0.3	11.5 ± 0.3	13.9 ± 0.2	17.2 ± 0.3	19.0 ± 0.3
S80	20.9 ± 0.4	16.6 ± 0.4	11.0 ± 0.3	15.9 ± 0.4	8.2 ± 0.3	12.9 ± 0.4	11.8 ± 0.2	12.5 ± 0.2	18.2 ± 0.2	8.3 ± 0.2	11.7 ± 0.4	12.9 ± 0.3	16.5 ± 0.3	18.5 ± 0.3	21.3 ± 0.4
L84	14.5 ± 0.3	12.3 ± 0.3	8.2 ± 0.5	12.8 ± 0.4	8.8 ± 0.3	12.6 ± 0.3	12.3 ± 0.4	9.8 ± 0.4	14.8 ± 0.5	9.3 ± 0.4	6.0 ± 0.3	6.0 ± 0.3	9.7 ± 0.3	13.9 ± 0.3	15.4 ± 0.3
L87	9.2 ± 0.4	6.3 ± 0.4	**4.5 ± 0.3**	9.3 ± 0.3	8.4 ± 0.5	15.9 ± 0.3	13.7 ± 0.3	9.3 ± 0.2	13.7 ± 0.2	6.0 ± 0.3	6.1 ± 0.4	5.8 ± 0.2	5.4 ± 0.4	9.6 ± 0.3	10.3 ± 0.4
I88	11.4 ± 0.3	10.3 ± 0.2	10.0 ± 0.3	14.9 ± 0.3	13.0 ± 0.3	19.5 ± 0.3	17.7 ± 0.4	13.9 ± 0.3	18.2 ± 0.4	10.8 ± 0.3	7.3 ± 0.4	9.2 ± 0.3	5.7 ± 0.2	6.2 ± 0.2	11.0 ± 0.2
V91	**5.8 ± 0.2**	6.2 ± 0.3	9.2 ± 0.3	12.5 ± 0.3	13.7 ± 0.3	19.7 ± 0.3	18.6 ± 0.3	13.7 ± 0.3	16.8 ± 0.3	8.6 ± 0.3	7.8 ± 0.4	**5.6 ± 0.2**	**5.7 ± 0.3**	5.4 ± 0.1	5.9 ± 0.2
A92	9.7 ± 0.2	10.4 ± 0.2	13.6 ± 0.4	17.8 ± 0.3	17.8 ± 0.3	24.2 ± 0.2	23.0 ± 0.3	18.5 ± 0.3	22.0 ± 0.4	12.1 ± 0.3	11.6 ± 0.4	10.24 ± 0.3	7.5 ± 0.5	8.7 ± 0.3	7.4 ± 0.1
A94	**4.5 ± 0.2**	6.5 ± 0.4	12.7 ± 0.6	15.0 ± 0.3	16.8 ± 0.3	23.1 ± 0.3	23.0 ± 0.3	17.9 ± 0.3	20.2 ± 0.3	12.4 ± 0.3	13.4 ± 0.4	9.2 ± 0.3	10.9 ± 0.4	9.8 ± 0.3	15.0 ± 0.3

Supplementary Table S5 I Intersubunit interactions in the domain-swapped TRAAK during 10 ns of MD simulations

The diagonal line, highlighted in gray, indicates the distance to the same residue in the neighboring subunit of the dimeric protein. Intersubunit distances are listed under the diagonal line and are written in black. Intrasubunit interactions which should be less relevant for the dimerization of the channel are listed over the diagonal line and written in a light grey. Distances are measured from a predefined carbon of the amino acid side chain, lacking also the hydrogen atoms, and thus a more remote cut-off of 6 Å was used (Supplementary Fig. S3 and Methods). Intersubunit distances under 6 Å are indicated in red. Nine putative intersubunit interactions were identified. Mean ± SDEV.

Supplementary References

Graddis, T.J., Myszka, D.G. & Chaiken, I.M. Controlled formation of model homo- and heterodimer coiled coil polypeptides. *Biochemistry* **32,** 12664-12671 (1993).

O'Shea, E.K., Lumb, K.J. & Kim, P.S. Peptide 'Velcro': design of a heterodimeric coiled coil. *Curr. Biol.* **3,** 658-667 (1993).
